# Effect of amaranth flatbread on maternal anthropometry during pregnancy: Evidence from a cluster-randomized controlled trial in Southern Ethiopia

**DOI:** 10.1371/journal.pone.0360480

**Published:** 2026-10-08

**Authors:** Amanuel Yoseph, Lakew Mussie, Mehretu Belayneh, Francisco Guillen-Grima

**Affiliations:** 1 School of Public Health, College of Medicine and Health Sciences, Hawassa University, Hawassa, Ethiopia; 2 Adare General Hospital, Hawassa City Administration, Hawassa, Ethiopia; 3 Department of Health Sciences, Public University of Navarra, Pamplona, Spain; 4 Group of Clinical Epidemiology, Area of Epidemiology and Public Health, Healthcare Research Institute of Navarre (IdiSNA), Pamplona, Spain; 5 CIBER in Epidemiology and Public Health (CIBERESP), Institute of Health Carlos III, Madrid, Spain; 6 Department of Preventive Medicine, Clinica Universidad de Navarra, Pamplona, Spain; Debre Markos University, ETHIOPIA

## Abstract

**Introduction:**

Maternal undernutrition and iron deficiency anemia (IDA) remain major public health challenges in Ethiopia, contributing to adverse pregnancy outcomes and poor maternal health. Amaranth, a nutrient-dense pseudocereal, has demonstrated potential to improve maternal nutritional status, yet evidence from rigorous trials is limited. To evaluate the effect of amaranth grain flatbread on maternal anthropometric indicators - mid-upper arm circumference (MUAC), body mass index (BMI), and gestational weight change (GWG) – as proxies for overall nutritional status among pregnant women in Hawela Lida district, Southern Ethiopia.

**Methods:**

A parallel-group, two-arm cluster randomized controlled trial was conducted from September 2024 to February 2025. Pregnant women with mild-to-moderate anemia, residing in Hawela Lida district for at least six months, were enrolled before 12 weeks of gestation and randomly allocated to receive either 200 g/day of amaranth-chickpea flatbread (intervention, n = 153) or maize flatbread (control, n = 153) for six months. Anthropometric measurements (MUAC, GWG, BMI) were collected at baseline and endline. Multilevel linear regression models adjusted for individual- and community-level covariates were used to estimate intervention effects.

**Results:**

Of the 306 participants, 293 (95.8%) completed the study. At endline, women in the intervention arm had significantly higher mean MUAC (26.1 ± 1.2 cm vs 24.3 ± 1.4 cm; adjusted β = 1.72, 95% CI: 1.48–1.96), BMI (23.8 ± 1.5 kg/m^2^ vs 21.9 ± 1.6 kg/m^2^; adjusted β = 1.89, 95% CI: 1.62–2.16), and GWG (8.4 ± 1.2 kg vs 6.1 ± 1.4 kg; adjusted β = 2.23, 95% CI: 1.92–2.54) compared to the control group. Nutritional improvements were consistent across subgroups, with no serious adverse events reported. Sensitivity analyses using categorical definitions of maternal nutritional status yielded results consistent with the primary analyses, confirming the robustness and public health relevance of the intervention effects.

**Conclusions:**

Daily consumption of amaranth grain flatbread significantly improved maternal anthropometric indicators, suggesting its potential as a sustainable nutritional intervention to enhance maternal nutritional status in resource-limited settings. These findings support integration of nutrient-rich traditional foods into maternal dietary programs.

## Introduction

Maternal undernutrition during pregnancy remains a pervasive global health challenge, disproportionately affecting low- and middle-income countries (LMICs). It is associated with adverse outcomes including intrauterine growth restriction, preterm birth, and increased maternal morbidity and mortality [1,2]. Despite global commitments to improving maternal nutrition, an estimated 32 million pregnant women worldwide are undernourished, with the highest burden concentrated in sub-Saharan Africa and South Asia [3]. Traditional metrics for maternal nutritional assessment such as body mass index (BMI), mid-upper arm circumference (MUAC), and gestational weight gain (GWG) – are widely used as proxies for energy and nutrient sufficiency [4]. These indicators not only predict short-term pregnancy outcomes but also intergenerational consequences for offspring health, underscoring their public health importance [5].

In Ethiopia, maternal undernutrition continues to be a critical determinant of poor maternal and neonatal outcomes. Recent national surveys suggest that approximately 22% of Ethiopian women of reproductive age have chronic energy deficiency, with significant regional variations [6]. In particular, the Sidama region reports persistently high rates of maternal anemia and undernutrition, exacerbated by limited dietary diversity and reliance on staple cereals such as maize [7]. While iron deficiency anemia has been extensively documented, maternal anthropometric deficiencies have received comparatively less attention, despite their direct relationship to maternal and child health [8]. Moreover, existing nutritional programs have primarily emphasized supplementation and fortification strategies, which are often costly, poorly sustained, and culturally incongruent [9]. Thus, there is an urgent need for food-based interventions that are affordable, acceptable, and nutritionally efficacious.

Although limited dietary diversity is a major contributor to maternal anaemia in Ethiopia, achieving broad dietary change during pregnancy is often constrained by socioeconomic and food-access factors [7]. In this study, amaranth flatbread was introduced as a nutrient-dense addition to usual diets rather than as a substitute for dietary diversity. Importantly, women in both the intervention and control groups received the same nutrition counselling, which emphasized maintaining dietary diversity, adhering to prescribed medications, and attending routine antenatal services in line with national guidelines. The only distinction between study arms was the type of flatbread provided, allowing the intervention to enhance nutrient intake without discouraging diversified food consumption.

Amaranth grain, an underutilized pseudo-cereal indigenous to Africa and Latin America, has garnered growing attention for its superior nutrient profile compared to traditional cereals [10]. It contains high-quality protein, bioavailable iron, calcium, and other essential micronutrients, and has lower phytate content when subjected to traditional processing methods such as soaking, fermentation, and germination [11]. Evidence from Ghana and Kenya suggests that amaranth-based interventions improve hemoglobin concentrations and reduce anemia in children and women [12,13]. However, data remain sparse on its effects on broader maternal anthropometric outcomes, including BMI, MUAC, and GWG, during pregnancy. To our knowledge, no published randomized trial has evaluated the impact of amaranth grain flatbread – a culturally appropriate dietary form – on these anthropometric markers among pregnant women in Ethiopia or comparable LMIC contexts. This represents a critical gap in the evidence base.

The rationale for examining anthropometric outcomes extends beyond micronutrient sufficiency. While iron deficiency anemia has been the dominant focus of maternal nutrition research, maternal energy reserves and macronutrient sufficiency as reflected in MUAC, BMI, and GWG – are equally vital for predicting maternal survival, delivery complications, and neonatal outcomes [14,15]. Furthermore, anthropometric measures are simple, inexpensive, and scalable tools that can be readily integrated into routine antenatal care in low-resource settings [16]. Demonstrating that amaranth-based dietary interventions can improve these indices would provide robust evidence for policy-makers seeking sustainable and culturally aligned nutrition solutions.

This study therefore aimed to investigate the effect of daily consumption of amaranth grain flatbread, compared to maize-based flatbread, on maternal anthropometric indicators including MUAC, BMI, and gestational weight change among pregnant women with mild to moderate anemia in the northern zone of the Sidama region, Ethiopia. By focusing on these broader indicators of maternal nutritional status, the study extends current knowledge beyond hematologic outcomes, addressing a significant research gap with direct implications for maternal health interventions in Ethiopia and similar LMIC contexts. We hypothesized that daily consumption of amaranth-chickpea flatbread during pregnancy would enhance maternal nutritional status, as reflected by improved MUAC, BMI, and gestational weight gain, compared with a maize-based flatbread.

## Methods

### Reporting and ethical considerations

This trial was reported in accordance with the Consolidated Standards of Reporting Trials (CONSORT) extension for cluster randomized controlled trials (found on Supplementary File 1 [S1 File]) [17]. The study was registered in the International Clinical Trials Registry (NCT06536153). No deviations from the original protocol occurred. Ethical approval was obtained from the Institutional Review Board at the College of Medicine and Health Sciences, Hawassa University (IRB/027/16). Support letters were secured from Hawassa University School of Public Health, the Sidama Region Health Bureau, and local kebele administrations. Community-level consent was obtained from local leaders before randomization, followed by written informed consent from each participant prior to enrollment. The participant information sheet and informed consent form are provided in S2 File.

### Study setting and design

The trial was conducted in Hawela Lida district, Sidama region, Ethiopia, between September 2024 and February 2025. The district has a predominantly agrarian economy, with enset, maize, coffee, khat, barley, and legumes as major crops. Health infrastructure includes 20 health posts and 4 health centers, with an estimated service coverage of 70% [18].

A parallel two-arm cluster randomized controlled trial design was applied. Entire *kebeles* (the smallest administrative units in Ethiopia) were randomized to either the intervention (amaranth grain flatbread) or control (maize flatbread) arm. All eligible women within a cluster received the same allocation throughout pregnancy.

### Study population and eligibility criteria

Pregnant women were eligible if they were: (i) <3 months’ gestation at enrollment, (ii) residents of the district for ≥6 months, and (iii) diagnosed with mild-to-moderate anemia based on hemoglobin and serum ferritin. Gestational age at enrollment was estimated using the last menstrual period (LMP) and, when available, confirmed by early ultrasound for women who reported having undergone the scan. Women were excluded if they had severe anemia (Hb < 7.0 g/dL), chronic illnesses (HIV/AIDS, tuberculosis, cancer), psychiatric disorders, history of recent blood transfusion, or recurrent malaria infection, hypotension, or were taking medications that could interact with vitamin K (such as anticoagulants). This ensured that participants with potential health risks related to the intervention were not enrolled, safeguarding their well-being throughout the study. Women with severe anemia identified at baseline were referred for immediate medical care.

### Randomization and sampling procedure

From 13 *kebeles* in the district, 10 were randomly selected. Stratified randomization was applied by urban/rural status to ensure balance. Using a computer-generated random allocation sequence, 5 *kebeles* were assigned to the intervention and 5 to the control arm by an independent biostatistician. Within each *kebele*, 31 eligible pregnant women were randomly selected following a census of households, yielding a total sample of 306 (153 per arm). The trial was designed as a cluster-randomized study, with each *kebele* (local administrative unit) serving as a cluster. The cluster size was set at 31 participants per kebele, based on our sample size calculation, which accounted for the expected effect size of the intervention, an intra-cluster correlation coefficient (ICC) of 0.03, a two-sided significance level of 5%, and 80% study power.

In *kebeles* where more than 31 eligible pregnant women were identified, only 31 participants were randomly selected to ensure equal cluster sizes across all *kebeles*. Within each selected *kebele,* all eligible pregnant women were first identified through a household census. From this complete list, 31 participants per *kebele* were randomly chosen using a computer-generated random number sequence by an independent biostatistician. This ensured that every eligible woman had an equal chance of selection and that cluster sizes remained consistent across *kebeles.* Maintaining consistent cluster sizes was essential to reduce bias and ensure comparability of outcomes across clusters. This approach preserves the statistical integrity of the cluster-randomized design while accommodating practical recruitment constraints.

### Masking (Blinding)

The study was conducted using a double-masked design. Participants, bread distributors, and outcome assessors were all unaware of the group allocation. To maintain masking, the amaranth and maize breads were prepared to be as similar as possible in appearance, texture, and packaging, and were delivered in identical containers without any identifying labels. Bread distribution and outcome assessment were carried out by separate teams, and assessors had no access to allocation information at any stage of the study. These measures were taken to minimise both performance and measurement bias.

### Intervention procedures

The amaranth and maize flatbreads used in this trial were prepared following standardized, locally feasible procedures to ensure both safety and nutritional integrity. The amaranth flatbread consisted of 70% amaranth grain and 30% chickpea, while the control maize flatbread consisted of 100% maize flour. All grains were sourced from certified local suppliers and inspected for quality and contamination prior to processing.

#### Processing.

Grains were cleaned, soaked, germinated, roasted, and fermented using traditional methods optimized to reduce antinutrients and enhance nutrient bioavailability [19,20]. The processed flours were then mixed with water and shaped into flatbreads of approximately 200 g per serving. Flatbreads were baked under controlled conditions and individually packaged in food-grade, sealed wrappers for daily distribution. The amaranth-chickpea and maize flatbreads were prepared locally in Hawela Lida district using traditional household processing techniques. These supplements were not commercially available. To ensure safety for consumption, microbiologic testing was conducted at the Hawassa University Food Science Laboratory, which included screening for Salmonella, E. coli, and other common contaminants.

#### Microbiologic safety testing.

Both flatbreads underwent microbiological testing before distribution, including total plate count, yeast and mold counts, and absence of Salmonella and Escherichia coli, in accordance with international food safety standards. No microbial contamination was detected, confirming that the flatbreads were safe for consumption.

#### Nutrient composition.

The macronutrient content (protein, carbohydrate, fat, and fiber) and key micronutrients (iron, calcium, zinc, vitamin A, and folate) were analyzed in triplicate at an accredited laboratory (Hawassa University and Ethiopian Public Health Institute Food Science Laboratory). Amaranth flatbread demonstrated higher protein, iron, calcium, and essential amino acid content compared to the maize flatbread, while energy content was comparable between the two. Detailed nutrient composition per 100 g of each flatbread is provided in S3 File.

#### Quality assurance.

Daily production was supervised by trained personnel to ensure uniformity in size, weight, and composition. Distribution was monitored to confirm adherence and proper handling at the household level. This approach ensures that the intervention was nutritionally consistent, safe, and reproducible, providing a robust foundation for evaluating its effects on maternal anthropometric outcomes.

### Outcome assessment

The primary outcomes for this analysis were maternal anthropometric indicators, assessed at baseline (<3 months’ gestation), midline (~28 weeks), and endline (~36 weeks or delivery):

- **MUAC:** measured on the left arm to the nearest 0.1 cm using a non-stretchable tape. A MUAC <23 cm was considered indicative of undernutrition.
- **Weight:** measured to the nearest 0.1 kg using a calibrated digital scale (Seca, Hamburg, Germany), with participants in light clothing and barefoot.
- **Height:** measured once at baseline to the nearest 0.1 cm using a portable stadiometer.
- **BMI:** calculated as weight (kg) divided by height (m²).
- **GWG:** defined as the difference between baseline and follow-up weights at midline and endline.

All anthropometric measurements were taken in duplicate, and averages were used. Data collectors were trained health professionals; inter-observer reliability tests showed a technical error of measurement <2%.

### Sample size

The sample size of 306 participants was originally calculated to detect differences in iron deficiency anemia prevalence [21]. This sample provided sufficient power (>80%) to detect clinically meaningful differences in MUAC (≥0.5 cm), BMI (≥0.5 kg/m²), and GWG (≥1.0 kg) between groups, accounting for the cluster design (ICC = 0.05).

### Data collection and quality assurance

A structured, pretested questionnaire captured socio-demographic, obstetric, and dietary information (found on S4 File). Data were collected electronically using Open Data Kit (ODK) software, with built-in validation checks. Supervisors reviewed data daily for completeness and consistency (see S5 File). Field staff received intensive training, and laboratory and anthropometric equipment were calibrated regularly. A pretest was conducted in the Boricha district, and feedback obtained from this exercise was used to further refine the data collection tool. Data completeness and internal consistency were checked daily throughout the data collection period. In addition, the principal investigator provided continuous oversight, closely monitoring data collection procedures and ensuring adherence to quality assurance standards.

### Statistical analysis

Analyses were conducted using Stata version 17 (StataCorp, College Station, TX, USA). Descriptive statistics summarized baseline characteristics by trial arm. Continuous anthropometric outcomes (MUAC, BMI, GWG) were analyzed using mixed-effects linear regression models with random intercepts to account for clustering at the *kebele* level. Models were adjusted for baseline values, maternal age, parity, education, and household food security. Model selection was guided by Akaike’s Information Criterion (AIC), Bayesian Information Criterion (BIC), log-likelihood, and intraclass correlation coefficients (ICC). Variables with p < 0.25 in bivariable analysis or established clinical relevance were included in the multivariable model. Interaction terms were used to assess effect modification, and multicollinearity was checked using variance inflation factors (VIF < 5). Statistical significance was set at *p* < 0.05. Primary intervention effects were estimated using mixed-effects linear regression models with random intercepts for *kebele*, adjusting for baseline outcome values and prespecified covariates, consistent with an ANCOVA framework for cluster trials. Sensitivity analyses were conducted using categorical definitions of MUAC, BMI, and GWG based on standard clinical cut-offs. Cluster-adjusted multivariable models produced results consistent with the primary analyses, confirming that the intervention’s effects were robust to alternative outcome definitions.

### Ethics statement

The Institutional Review Board (IRB) of the College of Medicine and Health Sciences at Hawassa University in Ethiopia examined and approved the study protocol (Reference: IRB/027/16). The Hawela Lida District Health Office and the Sidama Regional Health Bureau granted administrative approvals. Prior to recruitment, all participants received information regarding the study’s goals, methods, possible risks, and advantages. Prior to participation, all subjects provided written informed consent.

## Results

### Trial profile

The flow of participants is presented in Fig 1. Of the 528 pregnant women screened, 306 were enrolled and randomized equally to intervention (n = 153) and control (n = 153) arms. At the time of recruitment, the mean gestational age was 11.02 ± 3.21 weeks. By the endline assessment, 293 participants (95.75%) completed follow-up: 146 (95.4%) in the amaranth group and 147 (96.1%) in the maize group. Attrition was similar across study arms (4.6% vs. 3.9%).

**Fig 1:**
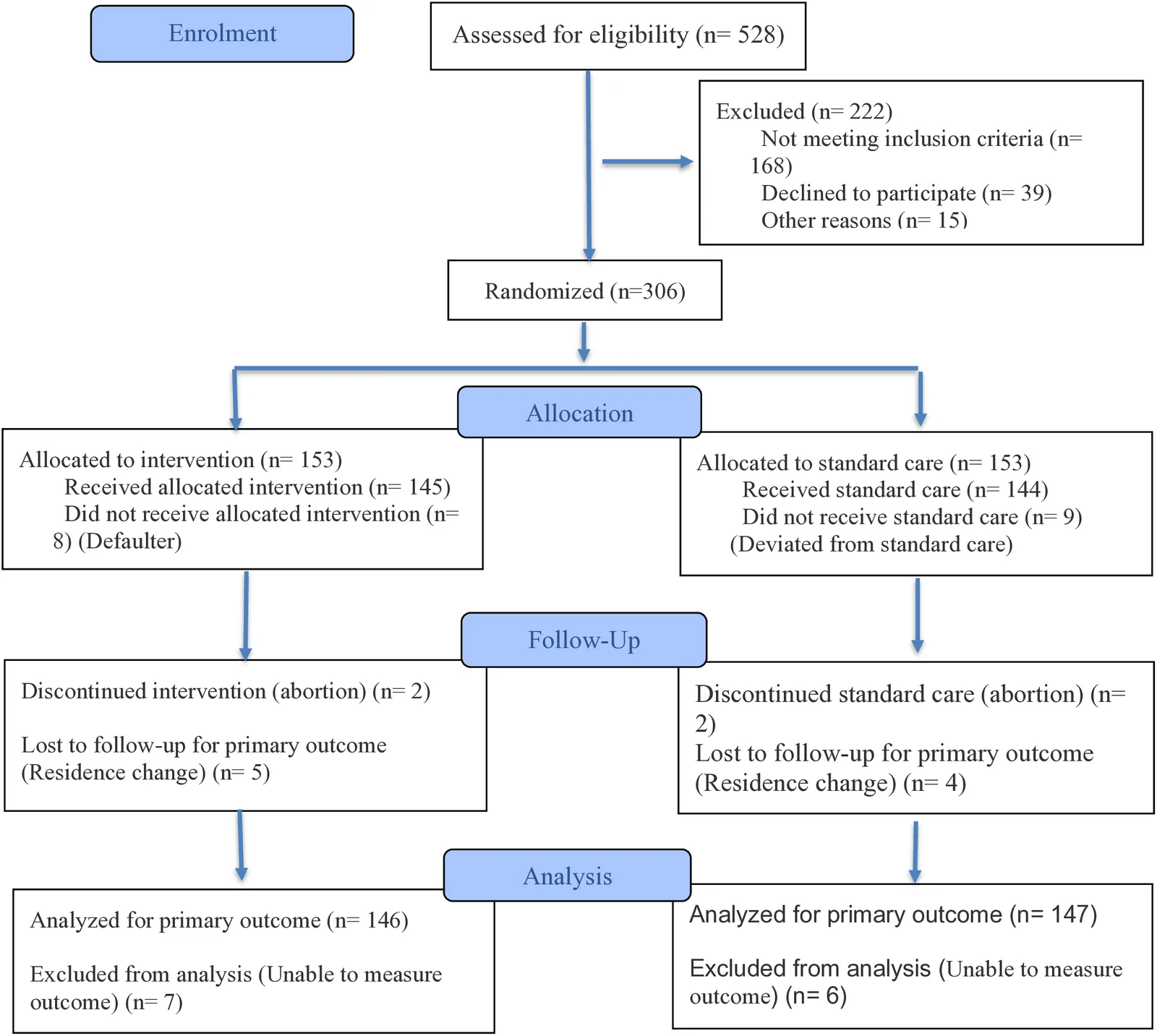
CONSORT 2025 flow diagram.

### Baseline characteristics

The baseline socio-demographic, economic, and obstetric characteristics were broadly comparable across groups (found on S6 File). Minor imbalances were observed in household wealth index, mass media use, and dietary diversity; these were adjusted for in subsequent analyses. Mean maternal MUAC, BMI, and weight at baseline were also similar between the two groups (Table 1).

**Table 1:** Baseline anthropometric characteristics of participants by study arm (descriptive statistics) in Hawale Lida district, Southern Ethiopia.

Variables	Intervention (Amaranth, n = 153)	Control (Maize, n = 153)	Total (N = 306)
MUAC (cm), mean ± SD	23.1 ± 2.1	23.0 ± 2.3	23.0 ± 2.2
BMI (kg/m²), mean ± SD	21.5 ± 2.4	21.6 ± 2.6	21.6 ± 2.5
Weight (kg), mean ± SD	53.7 ± 6.5	54.1 ± 6.9	53.9 ± 6.7
MUAC <23 cm, n (%)	42 (27.5)	45 (29.4)	87 (28.4)

Note: Values are presented for descriptive purposes only. No statistical tests were conducted at baseline, consistent with recommendations for cluster-randomised trials. BMI: Body mass index; MUAC: Mid upper arm circumference. Gestational age at enrollment was based on women-reported confirmed early ultrasound.

### Anthropometric outcomes at follow-up

At endline, women in the intervention group demonstrated significantly greater improvements in MUAC, BMI, and gestational weight gain (GWG) compared to the control group (Table 2). Endline anthropometric outcomes are presented descriptively by study arm, while intervention effects were formally estimated using cluster-adjusted mixed-effects models.

**Table 2:** Endline anthropometric outcomes by study arm (unadjusted descriptive statistics).

Outcomes	Intervention (n = 146)	Control (n = 147)	Mean Difference (95% CI)
MUAC (cm), mean ± SD	25.3 ± 2.2	23.7 ± 2.1	1.6 (1.1–2.1)
BMI (kg/m^2^), mean ± SD	23.4 ± 2.3	21.9 ± 2.5	1.5 (1.0–2.0)
Gestational weight gain (kg), mean ± SD	8.1 ± 2.9	5.9 ± 3.1	2.2 (1.5–2.9)
MUAC <23 cm, n (%)	18 (12.3)	43 (29.3)	ARR = 0.41 (0.23–0.73)

**Note:** Between-group differences were formally assessed using mixed-effects regression models accounting for clustering at the kebele level (see Table 4). BMI: Body mass index; MUAC: Mid upper arm circumference; GWG: Gestational weight gain; CI: confidence interval; SD: Standard deviation.

### Multivariable cluster adjusted analysis

After adjusting for baseline individuals and community-level covariates, the intervention group showed significantly better outcomes (Table 3).

**Table 3:** Adjusted effects of amaranth flatbread on maternal anthropometric outcomes (N = 293).

Outcome	Crude β (95% CI)	Adjusted β (95% CI)	*p*-value
MUAC (cm)	2.55 (1.11 - 4.89)	1.48 (1.12 - 1.84)	<0.001
BMI (kg/m^2^)	2.09 (1.65 - 3.7)	1.29 (0.95 - 1.63)	<0.001
Gestational weight gain (kg)	3.02 (1.77–6.45)	2.05 (1.51–2.59)	<0.001

**Note:** Confounders adjusted in the final models were wealth quintile, mass media use, age at first marriage and pregnancy, parity, iron rich food consumption, malaria infection, household food insecurity, dietary diversity, community-level wealth status, place of residence, and road accessibility; BMI: Body mass index; MUAC: Mid upper arm circumference; GWG: Gestational weight gain; β: beta coefficient and CI: confidence interval Pregnant women who consumed amaranth flatbread had a 59% lower risk of undernutrition (MUAC <23 cm) compared with controls (ARR = 0.41; 95% CI, 0.23–0.73).

### Anthropometric outcomes at follow-up (sensitivity analysis)

At endline, women in the intervention group showed significantly greater improvements in MUAC, BMI, and gestational weight gain (GWG) compared with the control group (Figs 2–5) and Table 4.

**Table 4:** Anthropometric outcomes at endline by category (N = 293).

Outcome	Category	Intervention (n = 146)	Control (n = 147)
Mid upper arm circumference (cm)	Green (≥23)	120 (82.2%)	104 (70.7%)
	Orange (21–22.9)	20 (13.7%)	32 (21.8%)
	Red (<21)	6 (4.1%)	11 (7.5%)
Transition (baseline → endline)	MAM→ Normal	59%	27%
Body mass index (kg/m^2^)	Underweight (<18.5)	12 (8.2%)	23 (15.6%)
	Normal (18.5–24.9)	115 (78.8%)	108 (73.5%)
	Overweight (25–29.9)	18 (12.3%)	14 (9.5%)
	Obese (≥30)	1 (0.7%)	2 (1.4%)
Gestational Weight Gain (kg)	Below recommended	29 (19.9%)	50 (34.0%)
	Within recommended	93 (63.7%)	61 (41.5%)
	Above recommended	24 (16.4%)	36 (24.5%)

**Note:** MUAC categories – green: normal nutritional status, orange: moderate acute malnutrition (MAM), red: severe acute malnutrition (SAM). BMI categories according to WHO cut-offs. GWG compared with WHO/IOM recommended ranges for gestational age.

**Fig 2:**
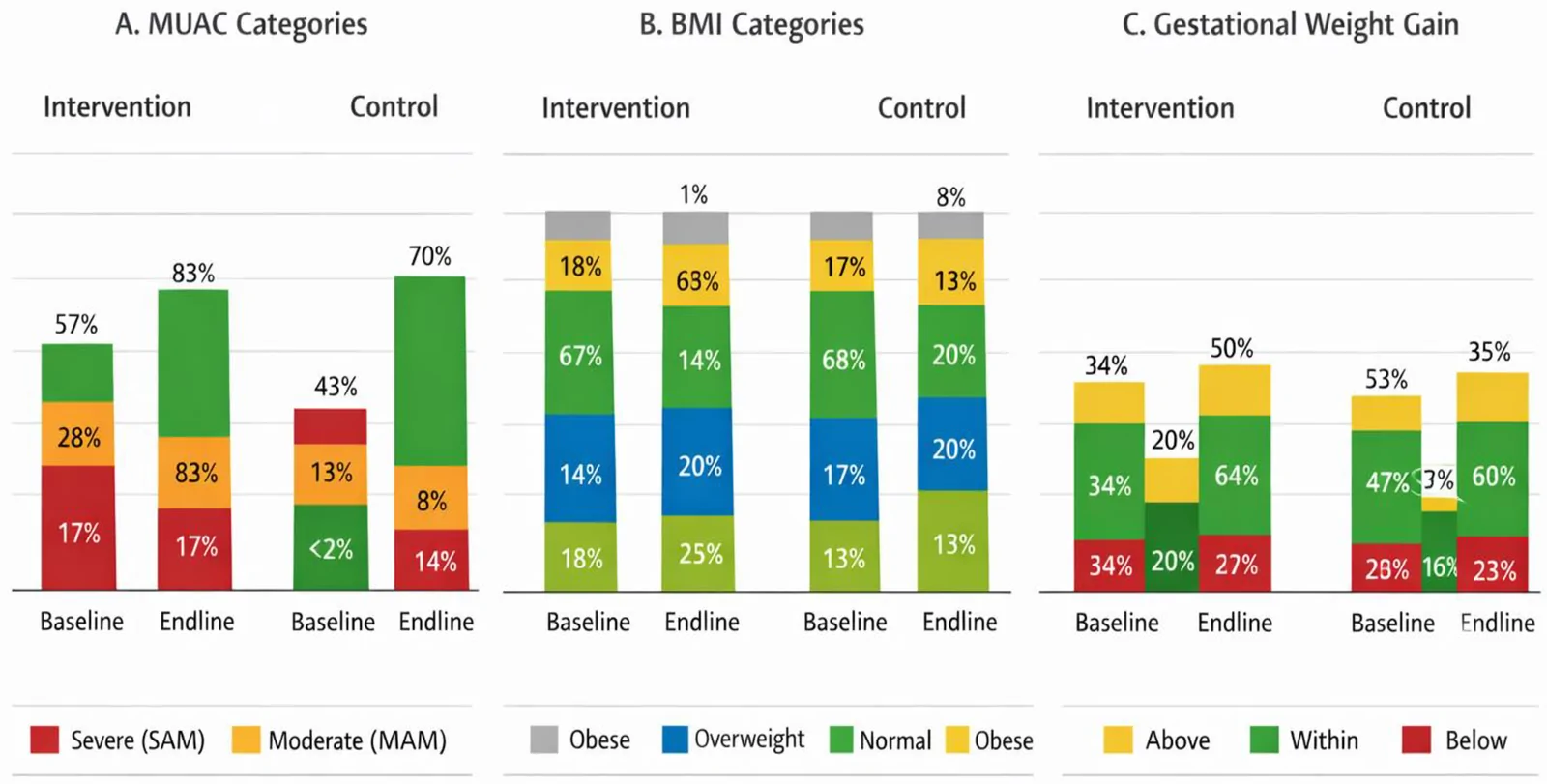
Changes in Maternal Nutritional Status from Baseline to Endline by Study Arm in Hawale Lida district, Southern Ethiopia.

**Fig 3:**
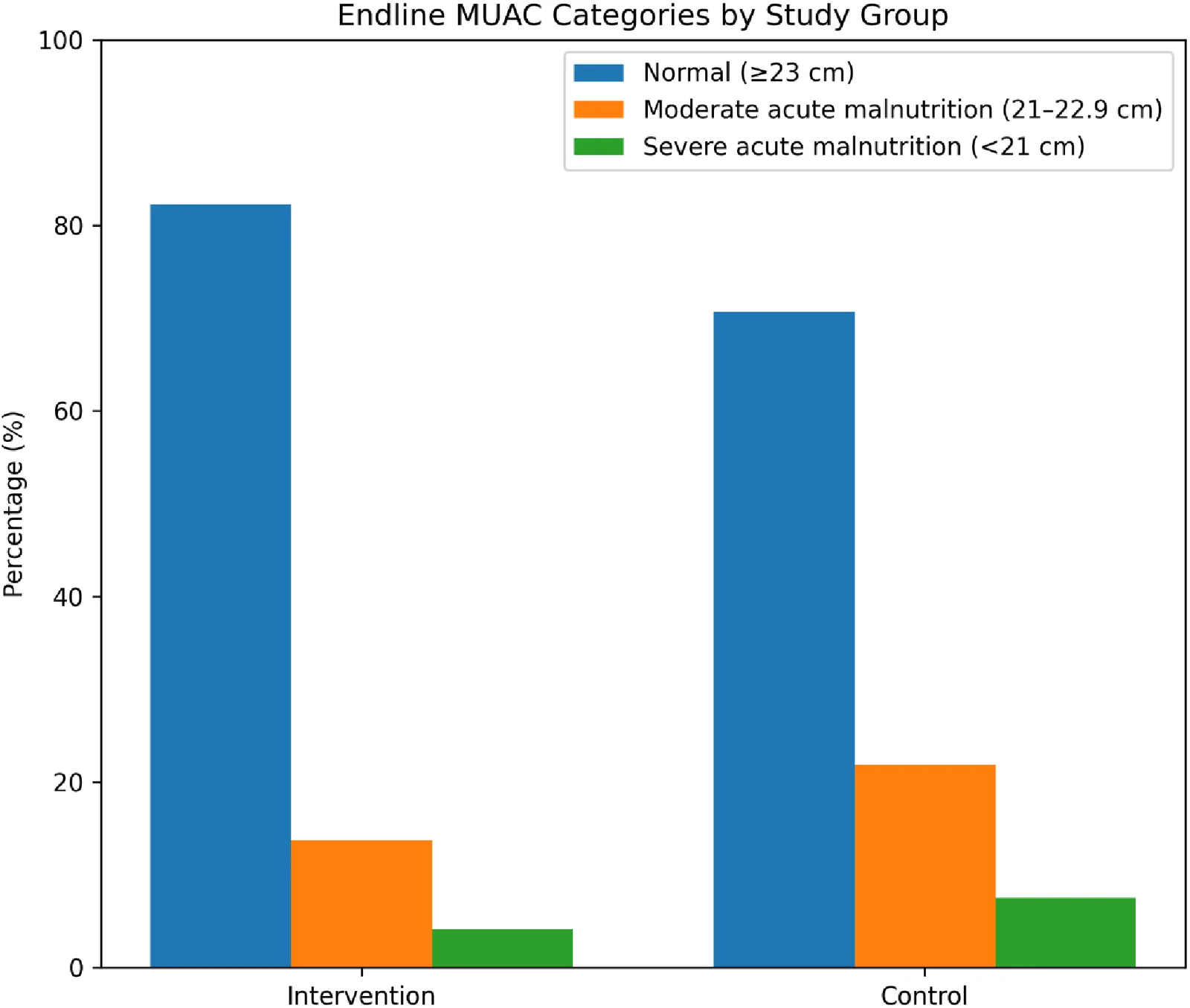
The endline mid upper arm circumference comparing intervention and control groups in Hawale Lida district, Southern Ethiopia.

**Fig 4:**
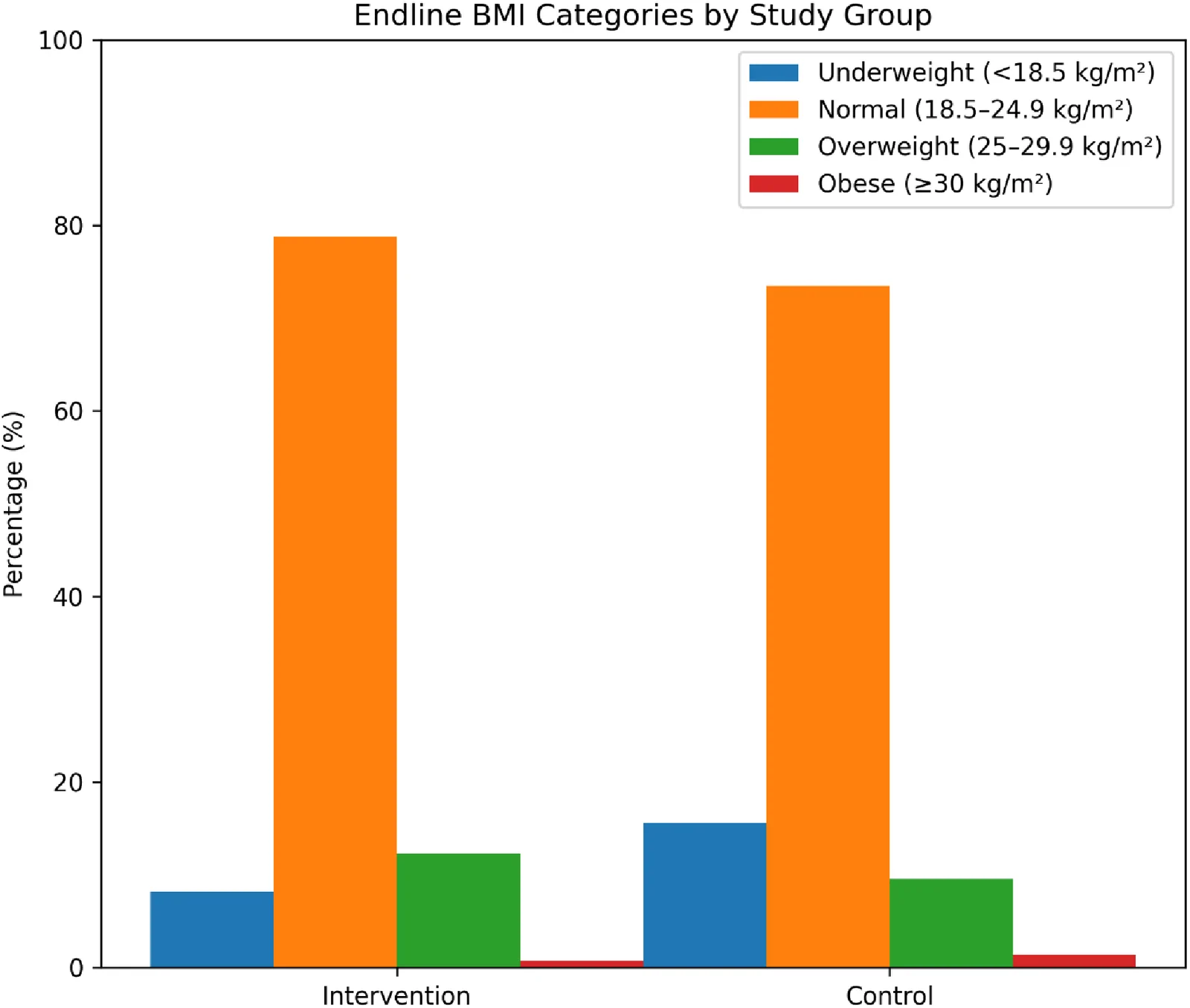
The endline body mass index comparing intervention and control groups in Hawale Lida district, Southern Ethiopia.

**Fig 5:**
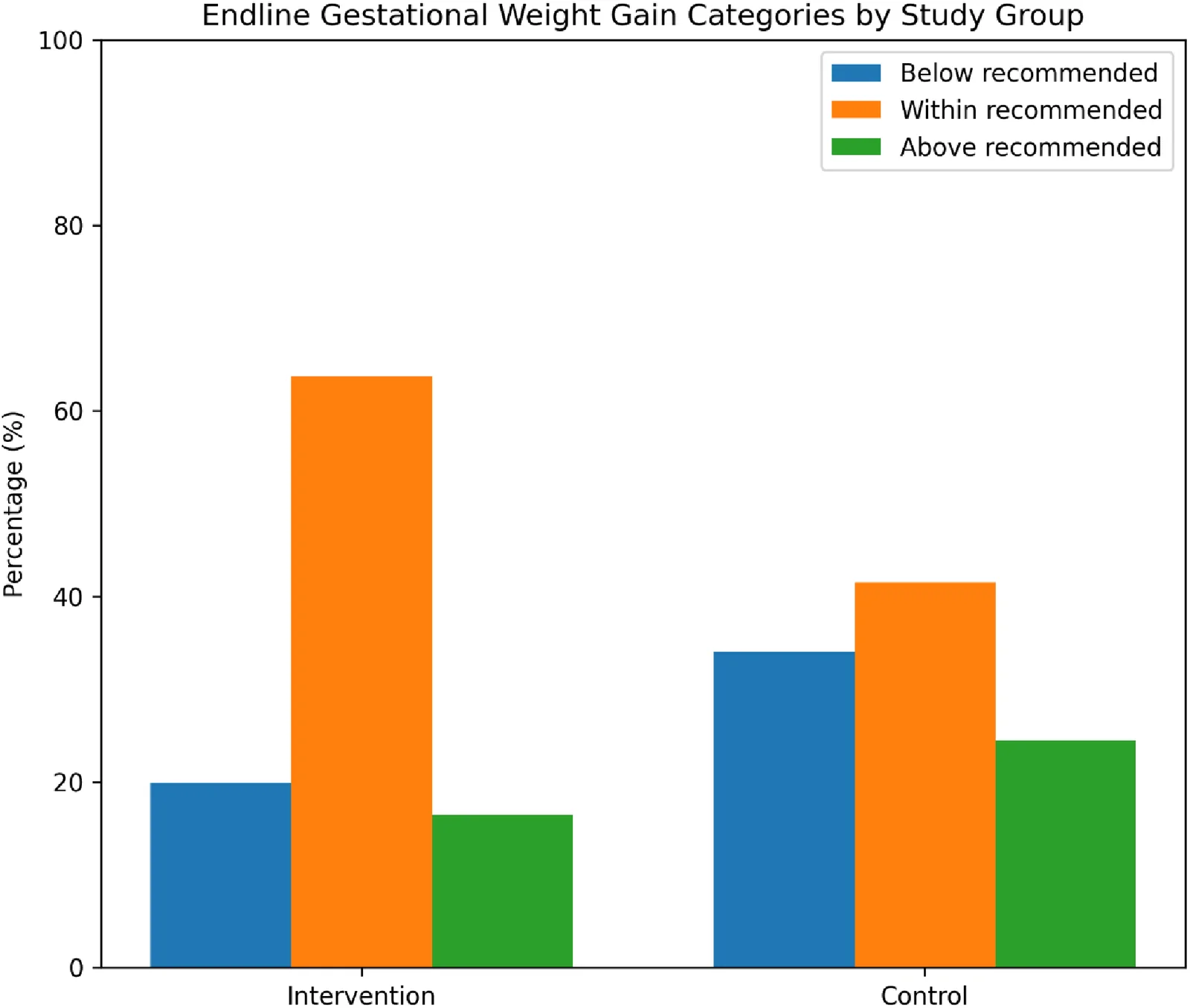
The endline gestational weight gain comparing intervention and control groups in Hawale Lida district, Southern Ethiopia.

### Mid upper arm circumference

MUAC was categorized using standard clinical cutoffs: < 21 cm (severe acute malnutrition [SAM]), 21–22.9 cm (moderate acute malnutrition [MAM]), and ≥23 cm (normal nutritional status). At baseline, 28.4% of participants had MUAC <23 cm. By endline, a substantially higher proportion of women in the intervention group fell within the normal range compared with controls, while fewer women remained in MAM or SAM categories, demonstrating the effectiveness of amaranth flatbread supplementation (Figs 2 and 3) and Table 4.

### Body mass index

BMI was classified using standard cut-offs: underweight (<18.5 kg/m^2^), normal weight (18.5–24.9), overweight (25–29.9), and obese (≥30) (Table 5). At baseline, 18% of participants were underweight across both arms. By endline, the proportion of participants achieving normal BMI was higher in the intervention group than in controls, reflecting meaningful improvements in maternal nutritional status (Figs 2 and 4).

**Table 5:** Effect of amaranth flatbread intervention on maternal nutritional outcomes at follow-up (sensitivity analysis using binary outcome categories) among pregnant women in Hawela Lida district, Sidama region, Ethiopia, 2025 (N = 293).

Variables	MUAC	CRR (95% CI)	ARR (95% CI)	
**MUAC**	**Undernutrition (<23 cm)**	**Normal (≥23 cm)**		
**Study group**				
Intervention (amaranth bread)	18 (12.3)	128 (87.7)	Ref	Ref
Comparator (maize bread)	43 (29.3)	104 (70.7)	0.32 (0.18–0.57)	0.35 (0.19–0.63)
	**BMI**			
	**Underweight (<18.5 kg/m²)**	**Normal (18.5–24.9 kg/m²)**		
**Study group**				
Intervention (amaranth bread)	36 (24.7)	110 (75.3)	Ref	Ref
Comparator (maize bread)	57 (38.8)	90 (61.2)	0.51 (0.31–0.85)	0.53 (0.32–0.87)
	**GWG**			
	**Within recommended range**	**Below/Above recommended**		
Study group				
Intervention (amaranth bread)	94 (64.4)	52 (35.6)	Ref	Ref
Comparator (maize bread)	60 (40.8)	87 (59.2)	2.56 (1.57–4.16)	2.42 (1.46–4.00)

**Note:** Confounders adjusted in the final models were wealth quintile, mass media use, age at first marriage and pregnancy, iron rich food consumption, malaria infection, household food insecurity, dietary diversity, community-level wealth status, place of residence, and road accessibility; Ref: reference group; ARR: Adjusted risk ratio; CRR: Crude risk ratio; CI: confidence interval; BMI: Body mass index; MUAC: Mid upper arm circumference; GWG: Gestational weight gain.

### Gestational weight gain

GWG was assessed against recommended ranges based on gestational age (WHO/IOM guidelines). At endline, 64% of participants in the intervention group achieved the recommended weight gain, compared with 41% in the control group. The proportion exceeding or falling below the recommended ranges is presented in Figs 2 and 5. This categorical approach emphasizes the clinical and public health relevance of the intervention, beyond population means (Table 4).

### Multivariable cluster-adjusted analyses

Multilevel multivariable Poisson regression analyses confirmed these findings. The intervention group had a 65–68% lower risk of maternal undernutrition (MUAC <23 cm; ARR = 0.35, 95% CI: 0.19–0.63) and a 47% lower risk of being underweight (BMI < 18.5 kg/m^2^; ARR = 0.53, 95% CI: 0.32–0.87) compared with controls. Women in the intervention group were also more than twice as likely to achieve recommended GWG (ARR = 2.42, 95% CI: 1.46–4.00) (Table 5).

Overall, these results demonstrate that daily consumption of nutrient-dense amaranth flatbread significantly improved maternal nutritional status and helped a greater proportion of women move from at-risk to normal categories, reinforcing the public health relevance of the intervention (found on S6 File).

### Subgroup Analyses by maternal age and nutritional status

To explore potential effect modification, stratified analyses were conducted by baseline nutritional status (MUAC <23 cm vs. ≥ 23 cm) and maternal age (adolescents <18 years vs. adults ≥18 years). Each potential effect modifier was entered individually into the final multivariable model as an interaction term with the intervention. None of the interaction terms reached statistical significance (all p > 0.05), indicating that the effect of the amaranth flatbread on maternal anthropometric outcomes was consistent across subgroups defined by maternal age and baseline nutritional status.

### Acceptability of the amaranth flatbread

Participant feedback indicated high acceptability of the amaranth flatbread. Over 90% of women reported consuming the flatbread consistently throughout the study period. Most participants described the taste and texture as pleasant, and nearly all expressed willingness to continue its consumption. No major complaints were reported, suggesting that the supplement is well tolerated and culturally acceptable in this population.

### Adverse effects monitoring

Participants were monitored regularly for any adverse events throughout the trial. Minor gastrointestinal discomforts were self-limiting and did not require medical intervention. No serious adverse events, including issues related to blood pressure or potential interactions with concurrent medications, were observed, indicating that the intervention was safe and well-tolerated by all participants (see S7 File).

### Adherence to the intervention

Adherence to the distributed breads was closely monitored throughout the trial. Bread distributors delivered the breads daily to participants’ homes, observing consumption and recording any missed doses, refusals, or deviations in a protocol adherence log maintained by the study coordinator. Based on these records, adherence remained high, with 95% of participants in both the intervention and control groups consuming the breads consistently. This monitoring approach ensured accurate tracking of intake and maintained the integrity of the intervention (found on S8 File). We conducted the study according to the approved study protocol, which is provided as S9 File.

### Random effects

Multilevel mixed-effects models with robust variance estimation showed significant clustering at the *kebele* level, with ICC values of 0.11 for MUAC, 0.21 for BMI, and 0.15 for GWG. Model fit improved with adjustment for covariates.

## Discussion

This cluster randomized controlled trial assessed the effect of daily supplementation with amaranth flatbread on maternal anthropometric indicators, including MUAC, BMI, and GWG. To our knowledge, this is among the first rigorously designed community-based interventions in Ethiopia to investigate the potential of locally available biofortified grains in improving maternal nutritional status. Our findings demonstrate that amaranth flatbread consumption significantly improved MUAC, BMI, and GWG compared to the control maize flatbread, suggesting a protective effect against maternal undernutrition during pregnancy.

Maternal undernutrition remains a persistent public health challenge in low- and middle-income countries, particularly in sub-Saharan Africa, where poor dietary diversity, food insecurity, and high infection burdens converge to compromise nutritional status [22,23]. Ethiopia has documented high levels of maternal undernutrition, with nearly one-quarter of pregnant women having MUAC <23 cm, reflecting energy and protein deficiencies [24]. The present trial addresses this critical gap by testing an affordable and culturally acceptable dietary intervention. Amaranth, a pseudocereal traditionally cultivated in East Africa, is rich in high-quality protein, iron, calcium, and bioavailable micronutrients [25,26]. The significant improvements in anthropometric outcomes observed in our study support its utility as a functional food to address maternal nutritional inadequacies.

Our findings align with previous small-scale supplementation studies that reported improved weight gain and BMI among pregnant women who received high-protein or micronutrient-dense foods [27,28]. The observed 2.2 kg greater GWG in the amaranth group is clinically meaningful, given that inadequate weight gain is strongly associated with low birth weight, preterm birth, and intrauterine growth restriction [29]. Similarly, improvements in MUAC reflect better maternal energy reserves, which are predictive of both maternal and neonatal outcomes [30]. Importantly, the protective effect against undernutrition (59% lower risk of MUAC <23 cm) highlights the potential of amaranth-based dietary strategies to prevent maternal wasting during pregnancy, particularly in resource-limited rural settings.

The biological plausibility of our results is supported by the nutrient composition of amaranth. Unlike maize, amaranth provides higher levels of essential amino acids such as lysine, which are critical for maternal tissue accretion [31]. Furthermore, amaranth contains significant levels of iron, zinc, and calcium, nutrients that are often deficient in maize-based diets [32]. Improved nutrient intake likely explains the higher GWG and BMI gains observed in our intervention group. These findings highlight the importance of diversifying staple foods with nutrient-rich alternatives to address both macronutrient and micronutrient deficiencies in pregnancy. Sensitivity analyses using categorical definitions of maternal nutritional status yielded findings consistent with the primary analyses, reinforcing the robustness and public health relevance of the observed effects.

The study also carries programmatic and policy implications. Current maternal nutrition interventions in Ethiopia largely focus on iron-folic acid supplementation and nutrition counseling, with limited emphasis on improving dietary quality through locally available foods [33]. Our results suggest that integrating amaranth into community-level dietary interventions could represent a cost-effective and sustainable strategy to improve maternal nutrition. Unlike imported supplements, amaranth is culturally familiar, adaptable into traditional flatbreads, and resilient to drought, making it an attractive food-based solution in the context of climate vulnerability and food insecurity [34]. Scaling such interventions through women’s groups, health extension programs, and agricultural cooperatives could enhance both nutritional outcomes and local food system resilience.

### Limitations of the study

This study has several strengths, including its randomized controlled design, high adherence, and use of multiple anthropometric indicators, which together enhance the validity of the findings. Furthermore, the trial achieved minimal attrition, ensuring robust follow-up data. However, some limitations should be noted. First, anthropometric indicators, while widely used, may not fully capture changes in maternal body composition. Second, we did not measure dietary intake quantitatively, which may have introduced residual confounding from non-trial foods. Third, the follow-up was limited to the second and third trimesters, and therefore the long-term effects on birth outcomes remain to be assessed. Future research should include biochemical markers of nutrient status, neonatal outcomes, and cost-effectiveness analyses to inform scale-up.

Finally, although extensive measures were taken to maintain masking, complete blinding in food-based community interventions is inherently challenging. Both study arms received flatbreads presented as nutritionally beneficial and were accompanied by identical counselling; however, participants may have formed perceptions about the value of the food provided. Such perceptions could have influenced dietary behaviour, including possible substitution of other foods. While any expectancy effect would likely have been non-differential across groups, residual behavioural modification cannot be entirely excluded and should be considered when interpreting the findings.

Although we accounted for dietary diversity in our analyses, we did not collect detailed quantitative information on all foods consumed outside the trial intervention, and thus some residual confounding from non-trial food intake may remain. Although maternal outcomes were comprehensively assessed, neonatal outcomes such as birth weight and infant anthropometrics were not systematically collected for all participants. Consequently, we were unable to evaluate the direct effects of the intervention on fetal growth or neonatal health, which should be considered in future studies.

## Conclusion

In conclusion, daily consumption of amaranth flatbread during pregnancy significantly improved MUAC, BMI, and gestational weight gain, and reduced the risk of undernutrition among women in rural Sidama, Ethiopia. These findings support the integration of amaranth into maternal nutrition programs as a sustainable, culturally appropriate, and food-based strategy to address maternal undernutrition. Scaling up such interventions has the potential to improve maternal health and contribute to the broader agenda of achieving maternal and child nutrition targets in Ethiopia and beyond.

## Supporting information

S1 File CONSORT reporting guidelines for cluster RCTs checklist. (DOC)

S2 File Participant information sheet and consent form. (DOCX)

S3 File Nutritional composition of locally prepared diets. (DOCX)

S4 File English version study questionnaire. (DOC)

S5 File Dataset. (XLSX)

S6 File Detailed information from results sections. (DOCX)

S7 File Mild adverse reaction report. (DOCX)

S8 File Protocol adherence log. (DOCX)

S9 File Institutional Review Board-approved study protocol. (DOC)

## Abbreviations

AIC: Akaike information criteria
ARR: Adjusted risk ratios
BIC: Bayesian Information Criterion
BMI: body mass index
CI: confidence interval
CRR: Crude risk ratios
GWG: gestational weight gain
IOM: Institute of Medicine
ICC: Intra-class correlation coefficient
LMIC: low- and middle-income countries
MUAC: mid-upper arm circumference
SD: Standard deviation
WHO: World Health Organization
VIF: Variance inflation factor

## References

[1] Black RE, Victora CG, Walker SP, Bhutta ZA, Christian P, de Onis M, et al. Maternal and child undernutrition and overweight in low-income and middle-income countries. Lancet. 2013;382(9890):427–51. doi: 10.1016/S0140-6736(13)60937-X 23746772

[2] Christian P, Stewart CP. Maternal micronutrient deficiency, fetal development, and the risk of chronic disease. J Nutr. 2010;140(3):437–45. doi: 10.3945/jn.109.116327 20071652

[3] Phelps NH, Singleton RK, Zhou B, Heap RA, Mishra A, Bennett JE, et al. Worldwide trends in underweight and obesity from 1990 to 2022: A pooled analysis of 3663 population-representative studies with 222 million children, adolescents, and adults. The Lancet. 2024;403(10431):1027–50. 10.1016/S0140-6736(23)02750-2 PMC7615769 38432237

[4] Villar J, Ismail LC, Victora CG, Ohuma EO, Bertino E, Altman DG, et al. International standards for newborn weight, length, and head circumference by gestational age and sex: The Newborn Cross-Sectional Study of the INTERGROWTH-21st Project. The Lancet. 2014;384(9946):857–68. 10.1016/S0140-6736(14)60932-6 25209487

[5] Victora CG, Adair L, Fall C, Hallal PC, Martorell R, Richter L, et al. Maternal and child undernutrition: Consequences for adult health and human capital. The Lancet. 2008;371(9609):340–57. doi: 10.1016/s0140-6736(07)61692-4 PMC2258311 18206223

[6] EPHI. Mini demographic and health survey. Rockville, Maryland, USA: EPHI and ICF. 2019.

[7] Terefe AT, Aredo MK, Workagegnehu AM, Tesfaye WM. Effect of climate shocks on rural household welfare dynamics in Ethiopia: Evidence from panel data. Cogent Food & Agriculture. 2024;10(1). doi: 10.1080/23311932.2024.2356936

[8] Zerfu TA, Umeta M, Baye K. Dietary diversity during pregnancy is associated with reduced risk of maternal anemia, preterm delivery, and low birth weight in a prospective cohort study in rural Ethiopia. The American Journal of Clinical Nutrition. 2016;103(6):1482–8. 10.3945/ajcn.115.116798 27169832

[9] Bhutta ZA, Das JK, Rizvi A, Gaffey MF, Walker N, Horton S, et al. Evidence-based interventions for improvement of maternal and child nutrition: what can be done and at what cost? Lancet. 2013;382(9890):452–77. doi: 10.1016/S0140-6736(13)60996-4 23746776

[10] Rastogi A, Shukla S. Amaranth: A new millennium crop of nutraceutical values. Critical reviews in food science and nutrition. 2013;53(2):109–25. 10.1080/10408398.2010.517876 23072528

[11] Repo-Carrasco R, Espinoza C, Jacobsen S-E. Nutritional Value and use of the andean crops quinoa (Chenopodium quinoa) and Kañiwa (Chenopodium pallidicaule). Food Reviews International. 2003;19(1–2):179–89. doi: 10.1081/fri-120018884

[12] Mensah P, Tomkins A. Household-level technologies to improve the availability and preparation of adequate and safe complementary foods. Food Nutr Bull. 2003;24(1):104–25. doi: 10.1177/156482650302400106 12664529

[13] Konyole SO, Omollo SA, Kinyuru JN, Skau JKH, Owuor BO, Estambale BB, et al. Effect of locally produced complementary foods on fat-free mass, linear growth, and iron status among Kenyan infants: A randomized controlled trial. Matern Child Nutr. 2019;15(4):e12836. doi: 10.1111/mcn.12836 31045329 PMC6859993

[14] Kramer MS. The epidemiology of adverse pregnancy outcomes: An overview. J Nutr. 2003;133(5 Suppl 2):1592S-1596S. doi: 10.1093/jn/133.5.1592S 12730473

[15] Han Z, Mulla S, Beyene J, Liao G, McDonald SD, Knowledge Synthesis Group. Maternal underweight and the risk of preterm birth and low birth weight: A systematic review and meta-analyses. Int J Epidemiol. 2011;40(1):65–101. doi: 10.1093/ije/dyq195 21097954

[16] Tang AM, Chung M, Dong KR, Bahwere P, Bose K, Chakraborty R, et al. Determining a global mid-upper arm circumference cut-off to assess underweight in adults (men and non-pregnant women). Public Health Nutr. 2020;23(17):3104–13. doi: 10.1017/S1368980020000397 32799964 PMC10200499

[17] Campbell MK, Elbourne DR, Altman DG. CONSORT statement: extension to cluster randomised trials. BMJ. 2004;328(7441):702–8. 10.1136/bmj.328.7441.702 PMC381234 15031246

[18] Sidama regional health bureau. Annual regional health and health related reports and performance review. Hawassa, Ethiopia. 2024.

[19] Orsango AZ, Loha E, Lindtjørn B, Engebretsen IMS. Efficacy of processed amaranth-containing bread compared to maize bread on hemoglobin, anemia and iron deficiency anemia prevalence among two-to-five year-old anemic children in Southern Ethiopia: A cluster randomized controlled trial. PLoS One. 2020;15(9):e0239192. doi: 10.1371/journal.pone.0239192 32986748 PMC7521750

[20] Zebdewos A, Singh P, Birhanu G, Whiting S, Henry C, Kebebu A. Formulation of complementary food using amaranth, chickpea and maize improves iron, calcium and zinc content. Afr J Food Agri Nutr Develop. 2015;15(71):10290–304. doi: 10.18697/ajfand.71.15235

[21] Central Statistical Agency (CSA) [Ethiopia], ICF. Ethiopia Demographic and Health Survey 2016. Addis Ababa, Ethiopia; Rockville, MD: CSA and ICF. 2016.

[22] Yoseph A, Mussie L, Belayneh M. Individual, household, and community-level determinants of undernutrition among pregnant women in the northern zone of the Sidama region, Ethiopia: A multi-level modified Poisson regression analysis. PLoS One. 2024;19(12):e0315681. doi: 10.1371/journal.pone.0315681 39689095 PMC11651547

[23] Kikafunda JK, Lukwago FB, Turyashemererwa F. Anaemia and associated factors among under-fives and their mothers in Bushenyi district, Western Uganda. Public Health Nutr. 2009;12(12):2302–8. doi: 10.1017/S1368980009005333 19344541

[24] Ethiopian Statistical Service ESS, ICF. Ethiopia Demographic and Health Survey 2024–25: Key Indicators. Addis Ababa and Rockville, Maryland, USA: ESS and ICF. 2025.

[25] Yoseph A, Mussie L, Belayineh M, Aguinaga-Ontoso I, Guillen-Grima F, Mutwiri G. The determinants of coexisting anemia and undernutrition among pregnant women in Southern Ethiopia: A multi-level analysis. Healthcare (Basel). 2025;13(13):1495. doi: 10.3390/healthcare13131495 40648519 PMC12249147

[26] Muyonga JH, Andabati B, Ssepuuya G. Effect of heat processing on selected grain amaranth physicochemical properties. Food Sci Nutr. 2014;2(1):9–16. doi: 10.1002/fsn3.75 24804060 PMC3951545

[27] Adu-Afarwuah S, Lartey A, Zeilani M, Dewey KG. Acceptability of lipid-based nutrient supplements (LNS) among Ghanaian infants and pregnant or lactating women. Matern Child Nutr. 2011;7(4):344–56. doi: 10.1111/j.1740-8709.2010.00286.x 21496207 PMC6860738

[28] Ota E, Hori H, Mori R, Tobe-Gai R, Farrar D. Antenatal dietary education and supplementation to increase energy and protein intake. Cochrane Database Syst Rev. 2015;2015(6):CD000032. doi: 10.1002/14651858.CD000032.pub3 26031211 PMC12634316

[29] Institute of Medicine (US) and National Research Council (US) Committee to Reexamine IOM Pregnancy Weight Guidelines. Weight gain during pregnancy: reexamining the guidelines. Washington (DC): National Academies Press. 2009. doi: 10.17226/12584

[30] Tafesse T, Yoseph A, Mayiso K, Gari T. Factors associated with stunting among children aged 6-59 months in Bensa District, Sidama Region, South Ethiopia: Unmatched case-control study. BMC Pediatr. 2021;21(1):551. doi: 10.1186/s12887-021-03029-9 34872503 PMC8647487

[31] Martirosyan DM, Miroshnichenko LA, Kulakova SN, Pogojeva AV, Zoloedov VI. Amaranth oil application for coronary heart disease and hypertension. Lipids Health Dis. 2007;6:1. doi: 10.1186/1476-511X-6-1 17207282 PMC1779269

[32] Olawoye B, Kadiri O, Oluwajuyitan TD. Grain amaranth: processing, health benefits and applications. In Handbook of Cereals, Pulses, Roots, and Tubers. CRC Press;2021.pp. 221–34.

[33] Woldeyohannes M, Girma M, Petros A, Hussen A, Samuel A, Dinssa DA, et al. Ethiopia National Food and Nutrition Survey to inform the Ethiopian National Food and Nutrition Strategy: A study protocol. BMJ Open. 2023;13(4):e067641. doi: 10.1136/bmjopen-2022-067641 37185190 PMC10151871

[34] Calicioglu O, Flammini A, Bracco S, Bellù L, Sims R. The future challenges of food and agriculture: An integrated analysis of trends and solutions. Sustainability. 2019;11(1):222. doi: 10.3390/su11010222

