# Supplementary material for: Effect of amaranth flatbread on maternal anthropometry during pregnancy: Evidence from a cluster-randomized controlled trial in Southern Ethiopia
Source: PLoS One. 2026 Oct 8;21(10):e0360480. doi: 10.1371/journal.pone.0360480 (PMC13648999; doi:10.1371/journal.pone.0360480)
Supplement: Supplementary file 1 [file pone.0360480.s002.docx]

**Participant Information Sheet**

**Title of the Study:** Effect of Amaranth Flatbread on Maternal Anthropometry During Pregnancy: A Cluster-Randomized Trial in Hawela Lida District, Sidama Region, Ethiopia, 2025

**Principal Investigator: Amanuel Yoseph**

**Institution:** Hawassa University

**Contact:** +251 915683578

**1. Invitation to Participate**

You are being invited to take part in this research study because you are a pregnant woman living in the selected study area. Before you decide, it is important to understand why the research is being done and what it will involve. Please take time to read this information carefully and discuss it with your family or others if you wish. Participation is voluntary, and you may withdraw at any time without affecting your care or access to services.

**2. Purpose of the Study**

The purpose of this study is to examine whether daily consumption of amaranth flatbread improves the nutritional status of pregnant women compared with the usual maize bread. The study will measure changes in body weight, BMI, and mid-upper arm circumference (MUAC), among other health indicators.

**3. What Participation Involves**

If you agree to participate, you will be asked to:

- Receive a daily supply of flatbread (either amaranth or maize) delivered to your home by a trained distributor.
- Consume the provided bread as instructed.
- Allow trained study staff to measure your weight, height, MUAC, and other health indicators at baseline and follow-up.
- Complete short questionnaires on diet and health during home visits.

Participation will last for the duration of your pregnancy, with regular follow-ups conducted by the research team.

**4. Bread Distribution and Adherence Monitoring**

Bread will be delivered daily to your home by trained distributors, who will observe consumption and record any missed doses or refusals in a protocol adherence log. This monitoring ensures that the study accurately tracks your intake while maintaining safety and quality.

**5. Risks and Discomforts**

The breads are made from locally consumed grains and are generally considered safe. Some participants may experience mild gastrointestinal discomfort, which is usually temporary. If you experience any significant discomfort or health concerns, you can report them immediately to the study team.

**6. Benefits**

There may be direct benefits if the intervention improves your nutritional status and supports healthy pregnancy outcomes. Even if there is no direct benefit, your participation will help researchers understand better strategies for maternal nutrition that may benefit other women in your community in the future.

**7. Confidentiality and Data Protection**

All information collected during this study will be treated with the strictest confidentiality. Data entered the ODK tool will be stored on a secure local server and accessible only to authorized study personnel. No third parties will have access to your data.

Personal identifiers will be kept separate from study data, and all analyses will use coded, anonymized information. Your data will be used only for research purposes, including monitoring, analysis, and reporting of study findings.

**8. Voluntary Participation and Withdrawal**

Participation is entirely voluntary. You may choose not to participate or to withdraw at any time, without any negative consequences or impact on your access to health care.

**9. Compensation**

There is no financial compensation for participating in the study. All participants will, however, receive the daily bread for the study duration.

**10. Questions and Contact Information**

If you have any questions about the study or your rights as a participant, you can contact:

- **Principal Investigator:** Amanuel Yoseph
- **Ethics Committee Contact:** Hawassa University by +251 91 128 4973

**11. Consent Statement (To be Signed)**

I have read and understood the information provided above. I have had the opportunity to ask questions, and I voluntarily agree to participate in this study. I understand that I can withdraw at any time without penalty.

Participant Name / Signature: ___________________
Date: ___________________

Researcher Name / Signature: ___________________
Date: ___________________
