# Supplementary material for: Effect of amaranth flatbread on maternal anthropometry during pregnancy: Evidence from a cluster-randomized controlled trial in Southern Ethiopia
Source: PLoS One. 2026 Oct 8;21(10):e0360480. doi: 10.1371/journal.pone.0360480 (PMC13648999; doi:10.1371/journal.pone.0360480)
Supplement: Supplementary file 2 [file pone.0360480.s003.docx]

**Supplementary File 3.** Nutrient composition of amaranth and maize flatbreads used in the trial (per 100 g)

| **Component** | **Amaranth-Chickpea Flatbread** | **Maize Flatbread** | **Method of Analysis / Notes** |
| --- | --- | --- | --- |
| Ingredients | 70% amaranth grain, 30% chickpea | 100% maize flour | Based on standard recipes used in the study |
| Energy (kcal) | 360 | 355 | Calculated from macronutrients |
| Protein (g) | 11.8 | 6.5 | Kjeldahl method |
| Fat (g) | 5.0 | 3.0 | Soxhlet extraction |
| Carbohydrate (g) | 67.5 | 75.2 | Difference method |
| Dietary Fiber (g) | 7.2 | 2.5 | Enzymatic-gravimetric method |
| Iron (mg) | 6.8 | 2.3 | Atomic absorption spectrophotometry |
| Calcium (mg) | 170 | 40 | Atomic absorption spectrophotometry |
| Zinc (mg) | 3.9 | 1.5 | Atomic absorption spectrophotometry |
| Vitamin A (µg RE) | 22 | 4 | HPLC |
| Folate (µg) | 64 | 10 | Microbiological assay |
| Microbiologic Safety | Total plate count <10³ CFU/g; yeast/mold <10² CFU/g; Salmonella and E. coli absent | Same | ISO 4833-1, ISO 21527, ISO 6579 |
| Serving size | 200 g | 200 g | Daily intervention portion |
| Notes | Flours soaked, germinated, roasted, fermented to reduce phytates and enhance nutrient bioavailability | Traditional maize flour, processed similarly without amaranth | Nutrient bioavailability optimized by processing |
