# Supplementary material for: Effect of amaranth flatbread on maternal anthropometry during pregnancy: Evidence from a cluster-randomized controlled trial in Southern Ethiopia
Source: PLoS One. 2026 Oct 8;21(10):e0360480. doi: 10.1371/journal.pone.0360480 (PMC13648999; doi:10.1371/journal.pone.0360480)
Supplement: Supplementary file 3 [file pone.0360480.s006.docx]

**S6 File Table 1:** Socio-demographic attributes of the trial subjects (N = 293)

| **Variables** | **Intervention group (amaranth bread)** | **Comparator group (maize bread)** | **Total** | **p-value** |
| --- | --- | --- | --- | --- |
|  | N (%) | N (%) | N (%) |  |
| **Age of mothers (mean, SD)** | 25.91 ± 4.25 | 25.78 ± 4.55 | 25.85 ± 4.40 | 0.803 |
| **Ethnicity** |  |  |  | 0.751 |
| Sidama | 141 (95.9) | 142 (97.3) | 283 (96.6) |  |
| Others | 6 (4.1) | 4 (2.7) | 10 (3.4) |  |
| **Religions** |  |  |  | 0.034 |
| Protestant | 124 (84.4) | 135 (92.5) | 259 (88.4) |  |
| Orthodox | 6 (4.1) | 7 (4.8) | 13 (4.4) |  |
| Muslim | 7 (4.8) | 2 (1.4) | 9 (3.1) |  |
| Catholic | 10 (6.8) | 2 (1.4) | 12 (4.1) |  |
| **Mothers' education status** |  |  |  | 0.366 |
| No formal education | 45 (30.8) | 38 (25.9) | 83 (28.3) |  |
| Have formal education | 101 (69.2) | 109 (74.1) | 210 (71.7) |  |
| **Marital status** |  |  |  | 0.622 |
| Married | 144 (98.6) | 146 (99.3) | 290 (99.0) |  |
| Divorced | 2 (1.4) | 1 (0.7) | 3 (1.00) |  |
| **Husband education status** |  |  |  | 0.331 |
| No formal education | 19 (13.2) | 26 (17.8) | 45 (15.5) |  |
| Have formal education | 125 (86.8) | 120 (82.2) | 245 (84.5) |  |
| **Women's occupation status** |  |  |  | 0.570 |
| Housewife | 135 (92.5) | 133 (90.5) | 268 (91.5) |  |
| Merchant | 6 (4.1) | 10 (6.8) | 16 (5.5) |  |
| Government employee | 5 (3.4) | 4 (2.7) | 9 (3.1) |  |
| **Husband occupation status** |  |  |  | 0.634 |
| Farmer | 94 (65.3) | 88 (60.3) | 182 (62.8) |  |
| Merchant | 39 (27.1) | 43 (29.5) | 82 (28.3) |  |
| Daily labourer | 7 (4.9) | 7 (4.8) | 14 (4.8) |  |
| Government employee | 4 (2.8) | 8 (5.5) | 12 (4.1) |  |
| **Family size** |  |  |  | 0.408 |
| Small (1-5) | 118 (80.8) | 113 (76.9) | 231 (78.8) |  |
| Large (greater than 5) | 28 (19.2) | 34 (23.1) | 62 (21.2) |  |
| **Use of mass media** |  |  |  | 0.003 |
| No | 45 (30.8) | 70 (47.6) | 115 (39.2) |  |
| Yes | 101 (69.2) | 77 (54.2) | 178 (60.8) |  |
| **Wealth quintile** |  |  |  | 0.001 |
| Lowest | 31 (21.2) | 28 (19.0) | 59 (20.1) |  |
| Second | 20 (13.7) | 38 (25.9) | 58 (19.9) |  |
| Middle | 26 (17.8) | 33 (22.4) | 59 (20.1) |  |
| Fourth | 19 (13.0) | 40 (27.2) | 59 (20.1) |  |
| Highest | 50 (34.2) | 8 (5.4) | 58 (19.8) |  |

**Note:** P-values were calculated using the chi-square test for categorical variables and the independent samples t-test for continuous variables.

**S6 File Table 2:** Reproductive and obstetric health characteristics of the trial participants (N = 293)

| **Variables** | **Intervention group (amaranth bread)** | **Comparator group (maize bread)** | **Total** | **p-value** |
| --- | --- | --- | --- | --- |
|  | N (%) | N (%) | N (%) |  |
| **Age at first marriage** | 20.65 ± 2.76 | 21.41 ± 3.38 | 21.03 ± 3.11 | 0.037 |
| **Age at first pregnancy** | 21.86 ± 2.68 | 22.96 ± 3.65 | 22.41 ± 3.24 | 0.003 |
| **Total number of gravidities** |  |  |  | 0.374 |
| 1 | 45 (39.5) | 34 (32.4) | 79 (36.1) |  |
| 2-4 | 60 (52.6) | 65 (61.9) | 125 (57.1) |  |
| ≥5 | 9 (7.9) | 6 (5.7) | 15 (6.8) |  |
| **Previous history of abortions** |  |  |  | 0.243 |
| No | 142 (97.3) | 139 (94.6) | 281 (95.9) |  |
| Yes | 4 (2.7) | 8 (5.4) | 12 (4.1) |  |
| **Total number of deliveries** |  |  |  | 0.761 |
| 1 | 25 (27.8) | 24 (26.4) | 49 (27.1) |  |
| 2-4 | 59 (65.6) | 63 (69.2) | 122 (67.4) |  |
| ≥5 | 6 (6.7) | 4 (4.4) | 10 (5.5) |  |
| **Infection during the current pregnancy** |  |  |  | 0.636 |
| No | 124 (84.9) | 121 (82.3) | 245 (83.6) |  |
| Yes | 22 (15.1) | 26 (17.7) | 48 (16.4) |  |
| **Previous history of stillbirth** |  |  |  | 0.661 |
| No | 144 (98.6) | 144 (98.0) | 288 (98.3) |  |
| Yes | 2 (1.4) | 3 (2.0) | 5 (1.7) |  |
| **Previous history of neonatal death** |  |  |  | 0.657 |
| No | 144 (98.6) | 144 (98.0) | 288 (98.3) |  |
| Yes | 2 (1.4) | 3 (2.0) | 5 (1.7) |  |
| **Last pregnancy planned** |  |  |  | 0.490 |
| No | 16 (11.0) | 20 (13.6) | 36 (12.3) |  |
| Yes | 130 (89.0) | 127 (86.4) | 257 (87.7) |  |
| **Encountered obstetric danger signs during last pregnancy** |  |  |  | 0.932 |
| No | 134 (91.8) | 134 (91.2) | 268 (91.5) |  |
| Yes | 12 (8.2) | 13 (8.8) | 25 (8.5) |  |

**Note:** P-values were calculated using the chi-square test for categorical variables and the independent samples t-test for continuous variables.

**S6 File Table 3:** Endline anthropometric outcomes by study arm (unadjusted descriptive statistics).

| **Outcomes** | **Intervention (n=146)** | **Control (n=147)** | **Mean Difference (95% CI)** | **p-value** |
| --- | --- | --- | --- | --- |
| MUAC (cm), mean ± SD | 25.3 ± 2.2 | 23.7 ± 2.1 | 1.6 (1.1–2.1) | <0.001 |
| BMI (kg/m²), mean ± SD | 23.4 ± 2.3 | 21.9 ± 2.5 | 1.5 (1.0–2.0) | <0.001 |
| Gestational weight gain (kg), mean ± SD | 8.1 ± 2.9 | 5.9 ± 3.1 | 2.2 (1.5–2.9) | <0.001 |

**Note:** Between-group differences were formally assessed using mixed-effects regression models accounting for clustering at the *kebele* level (see Table 4). BMI: Body mass index; MUAC: Mid upper arm circumference; GWG: Gestational weight gain; CI: confidence interval; SD: Standard deviation.

**S6 File Table 4:** Effect of amaranth flatbread intervention on maternal nutritional outcomes at follow-up (sensitivity analysis using binary outcome categories) among pregnant women in Hawela Lida district, Sidama region, Ethiopia, 2025 (N = 293)

| **Variables** | **MUAC** | | **CRR (95% CI)** | **ARR (95% CI)** |
| --- | --- | --- | --- | --- |
| **MUAC** | **Undernutrition** | **Normal** |  |  |
| **Study group** |  |  |  |  |
| Intervention (amaranth bread) | 18 (12.3) | 128 (87.7) | Ref | Ref |
| Comparator (maize bread) | 43 (29.3) | 104 (70.7) | 0.32 (0.18–0.57) | 0.35 (0.19–0.63) |
|  | **BMI** | |  |  |
|  | **Underweight** | **Normal** |  |  |
| **Study group** |  |  |  |  |
| Intervention (amaranth bread) | 36 (24.7) | 110 (75.3) | Ref | Ref |
| Comparator (maize bread) | 57 (38.8) | 90 (61.2) | 0.51 (0.31–0.85) | 0.53 (0.32–0.87) |
|  | **GWG** | |  |  |
|  | **Within the recommended range** | **Below/Above recommended** |  |  |
| Study group |  |  |  |  |
| Intervention (amaranth bread) | 94 (64.4) | 52 (35.6) | Ref | Ref |
| Comparator (maize bread) | 60 (40.8) | 87 (59.2) | 2.56 (1.57–4.16) | 2.42 (1.46–4.00) |

**Note:** Confounders adjusted in the final models were wealth quintile, parity, mass media use, age at first marriage and pregnancy, iron rich food consumption, malaria infection, household food insecurity, dietary diversity, community-level wealth status, place of residence, and road accessibility; Ref: reference group; ARR: Adjusted risk ratio; CRR: Crude risk ratio; CI: confidence interval; BMI: Body mass index; MUAC: Mid upper arm circumference; GWG: Gestational weight gain.
