# Supplementary material for: Effect of amaranth flatbread on maternal anthropometry during pregnancy: Evidence from a cluster-randomized controlled trial in Southern Ethiopia
Source: PLoS One. 2026 Oct 8;21(10):e0360480. doi: 10.1371/journal.pone.0360480 (PMC13648999; doi:10.1371/journal.pone.0360480)
Supplement: Supplementary file 4 [file pone.0360480.s007.docx]

**Supplementary File 7:** Reported Adverse Events During the Intervention (N = 293)

| **Adverse Event** | **Intervention (n = 146)** | **Control (n = 147)** | **Total (N = 293)** | **Notes** |
| --- | --- | --- | --- | --- |
| Minor gastrointestinal discomfort (bloating, mild diarrhea) | 8 (5.5%) | 5 (3.4%) | 13 (4.4%) | Self-limiting, resolved without treatment |
| Transient rash or skin irritation | 3 (2.1%) | 2 (1.4%) | 5 (1.7%) | Mild, no medical intervention required |
| Mild nausea | 4 (2.7%) | 3 (2.0%) | 7 (2.4%) | Self-limiting |
| Others (e.g., mild abdominal cramps) | 2 (1.4%) | 1 (0.7%) | 3 (1.0%) | Resolved spontaneously |
