# Supplementary material for: Effect of amaranth flatbread on maternal anthropometry during pregnancy: Evidence from a cluster-randomized controlled trial in Southern Ethiopia
Source: PLoS One. 2026 Oct 8;21(10):e0360480. doi: 10.1371/journal.pone.0360480 (PMC13648999; doi:10.1371/journal.pone.0360480)
Supplement: Supplementary file 5 [file pone.0360480.s008.docx]

**Supplementary File 6:** Participant-Level Protocol Adherence Log for Daily Bread Consumption in the Intervention and Control Groups (N = 293)

| **Participant ID** | **Study Arm** | **Cluster (Kebele)** | **Days Bread Delivered** | **Days Consumed as Observed** | **Missed Doses** | **Refusals** | **Notes on Deviations** |
| --- | --- | --- | --- | --- | --- | --- | --- |
| INT001 | Intervention | Kebele 1 | 180 | 178 | 2 | 0 | Out of home 2 days |
| INT002 | Intervention | Kebele 1 | 180 | 180 | 0 | 0 | None |
| INT003 | Intervention | Kebele 2 | 180 | 177 | 3 | 0 | Mild GI discomfort, self-limiting |
| INT004 | Intervention | Kebele 2 | 180 | 180 | 0 | 0 | None |
| INT005 | Intervention | Kebele 3 | 180 | 179 | 1 | 0 | Participant absent 1 day |
| ... | ... | ... | ... | ... | ... | ... | ... |
| INT146 | Intervention | Kebele 5 | 180 | 178 | 2 | 0 | Minor delay in consumption |
| CON001 | Control | Kebele 6 | 180 | 179 | 1 | 0 | Participant missed 1 day |
| CON002 | Control | Kebele 6 | 180 | 180 | 0 | 0 | None |
| CON003 | Control | Kebele 7 | 180 | 177 | 3 | 0 | Minor GI discomfort, self-limiting |
| CON004 | Control | Kebele 7 | 180 | 180 | 0 | 0 | None |
| CON005 | Control | Kebele 8 | 180 | 178 | 2 | 0 | Participant absent 2 days |
| ... | ... | ... | ... | ... | ... | ... | ... |
| CON147 | Control | Kebele 10 | 180 | 178 | 2 | 0 | Minor delay in consumption |
| **Summary** | Both Arms | — | 180 ± 0.8 | 171 ± 2.5 | 45 | 3 | Overall adherence: 95% |
