# Supplementary material for: Effect of amaranth flatbread on maternal anthropometry during pregnancy: Evidence from a cluster-randomized controlled trial in Southern Ethiopia
Source: PLoS One. 2026 Oct 8;21(10):e0360480. doi: 10.1371/journal.pone.0360480 (PMC13648999; doi:10.1371/journal.pone.0360480)
Supplement: Supplementary file 7 [file pone.0360480.s004.doc]

## English version questionnaire

***Effect of Amaranth Grain Flatbread on Maternal Anthropometric Indicators During Pregnancy: Evidence from a Cluster-Randomized Trial in Ethiopia***

**PART I: General Information**

01. Name of *kebeles*: ____________

02. House number ____________________

03. Individual ID __________________

04. Date of interview: ____________time interview started __________ ended at ____________

05. Respondent available on:

1. 1^st^ visit.

2. 2^nd^ visit.

3. 3^rd^ visit.

**Part I: Socio-demographic characteristics of study participants**

**Instruction:** First, tell interviewee that you are going to ask her questions about herself. Second, properly explain to the study participant each question to elicit genuine response from the respondents. Then, request her to correctly respond to the questions. Please write the responses based on the respondent’s genuine responses (in field to be filled by data collectors).

| S.no | Questions | Response categories | Skip to | Remark |
| --- | --- | --- | --- | --- |
| 101 | What is your age (in completed years)? | __________ years |  |  |
| 102 | To which ethnic group do you belong? | 1. Sidama 2. Amhara 3. Oromo 4. Gurage 5. Wolayita 88.Other, specify_____ |  |  |
| 103 | What is your religious affiliation?  (encircle the response) | 1. Protestant Christian  2. Orthodox Christian  3. Catholic  4. Muslim  5.Other (specify) __________ |  |  |
| 104 | What is your highest educational level you have completed? | 1. Cannot read and write  2. Can read and write only  3. Primary education (1-8)  4. Secondary education (9-12)  5. College diploma  6. College/university degree and above |  |  |
| 105 | What is your occupation? | 1. Housewife  2. Farmer  3. Governmental employee  4. Merchant  5. Housemaid  6. Other (specify)……………… |  |  |
| 106 | What is your marital status? | 1. Not ever married…………….  2. Married  3. Divorced  4. Separated  5. Widowed  6. Cohabiting | Q110 |  |
| 107 | What is your husbands age in complete years? | _______________ |  |  |
| 108 | What is your husband’s occupation? | 1. Governmental employee  2. Merchant  3. Farmer  4. Daily labourer  5. NGO employee  6. Private organization employee  7. Other (specify)……………… |  |  |
| 109 | What is your husband’s educational status? | 1. Cannot read and write  2. Can read and write only  3. Primary education (1-8)  4. Secondary education (9-12)  5. College diploma  6. College/university degree and above |  |  |
| 110 | What is your family size? (In number) | ________ |  |  |
| 111 | Which social media do you use? | 1. Listens the radio 2. Watches the television 3. Reads the newspaper 4. All 5. Others |  |  |

Part II: Household wealth index of study participants

| S.no | Questions | Response categories | Skip to | Remark |
| --- | --- | --- | --- | --- |
| 201 | What is the main source of drinking water for members of your household? | 1. Piped water 2. Tube well or Borehole 3. Protected well 4. Unprotected well 5. Protected spring 6. Unprotected spring 7. Rainwater 8. Surface water (River/dam) 9. Lake/pond/stream/canal 10. Bottled water |  |  |
| 202 | What is the main source of water used by your household for other purposes such as cooking and handwashing? | 1. Piped water 2. Tube well or Borehole 3. Protected well 4. Unprotected well 5. Protected spring 6. Unprotected spring 7. Rainwater 8. Surface water (River/dam) 9. Lake/pond/stream/canal 10. Bottled water |  |  |
| 203 | Where is that water source located? | 1. In own dwelling 2. In own yard/plot 3. Elsewhere |  |  |
| 204 | How long does it take to go there, get water, and come back? | Minutes . . . . . . . . . . . . . . ..  998. Don’t know |  |  |
| 205 | In the past two weeks, was the water from this source not available for at least one full day? | 1. Yes 0. No   998. Don’t know |  |  |
| 206 | Do you do anything to the water to make it safer to drink? | 1. Yes 0. No   998. Don’t know |  |  |
| 207 | What kind of toilet facility do members of your household usually use?  IF NOT POSSIBLE TO DETERMINE, ASK PERMISSION TO OBSERVE THE FACILITY. | 1. Flush or pour flush toilet 2. Ventilated improved pit latrine 3. Pit latrine with slab 4. Pit latrine without slab/open pit 5. Compositing toilet 6. Bucket toilet 7. Hanging toilet 8. No facility/bush/field |  |  |
| 208 | Do you share this toilet facility with other households? | 1. Yes 0. No |  |  |
| 209 | Including your own household, how many households use this toilet facility? | 998. Don’t know |  |  |
| 210 | Where is this toilet facility located? | 1. In own dwelling  2. In own yard/plot  3. Elsewhere |  |  |
| 211 | What type of fuel does your household mainly use for cooking? | 1.Electricity  2. Liquefied petroleum gas  3. Natural gas  4.Biogas  5.Keresone  6. Charcoal  7. Wood  8. Straw/shrubs/grass  9. Agricultural crop  10. Animal dung  11.No food cooked in household |  |  |
| 212 | Is the cooking usually done in the house, in a separate building, or outdoors? | 1. In the house  2. In a separate building  3. Outdoors  4. Others (specify)……………… |  |  |
| 213 | Do you have a separate room which is used as a kitchen? | 1. Yes  0. No |  |  |
| 214 | Who is the owner of the house? | 1. Me 2. Rental 3. Family 4. Relative 5. Others (specify)…………... |  |  |
| 215 | How many rooms in this household are used for sleeping? | -------------------------- |  |  |
| 216 | Main material of the roof of the house? | 1. Natural roofing (no roof, mud, and sod) 2. Rudimentary roofing (rustic mat/plastic sheet, reed/bamboo, wood planks, and cardboard) 3. Finished roofing (metal/corrugated iron, wood, calamine/cement, ceramic tiles, roofing shingles) |  |  |
| 217 | Main material of the floor of the house? | 1. Natural floor (Earth/sand, dung) 2. Rudimentary floor (wood planks, and palm/bamboo) 3. Finished floor (parquet or polished wood, vinyl or asphalt strips/ plastic tiles, cement, ceramic tiles, carpet) |  |  |
| 218 | Main material of the floor of the house? | 1. Natural walls (no walls, cane/palm/trunks/bamboo/reef, dirt) 2. Rudimentary walls (bamboo with mud, stone with mud, uncovered adobe, plywood, cardboard, and reused wood) 3. Finished floor (cement, stone with lime/cement, bricks, cement blocks, covered adobe, wood planks/shingles) |  |  |
| 219 | Does this household own any livestock, herds, other farm animals, or poultry? | 1.Yes  0.No | 222 |  |
| 220 | How many of the following animals does this household own?  IF NONE, RECORD ‘00’.  IF 95 OR MORE, RECORD ‘95’.  IF UNKNOWN, RECORD ‘98’.  a) Milk cows, oxen or bulls?  b) other cattle?  c) Horses/donkeys/mules?  d) Camels?  e) Goats?  f) Sheep?  g) Chickens/poultry?  h) Beehives? | a) Milk cows, oxen or bulls………  b) other cattle………………….  c) Horses/donkeys/mules……….  d) Camels……………………...  e) Goats………………………  f) Sheep………………………….  g) Chickens/poultry…………….  h) Beehives……………………. |  |  |
| 221 | Do you have separate rooms for cattle? | 1.Yes  0.No |  |  |
| 222 | Does any member of this household own any agricultural land? | 1.Yes  0. No | 224 |  |
| 223 | How many hectares of agricultural land do members of this household own? | ---------------------------hectares |  |  |
| 224 | Does your household have:   a) Electricity? b) A radio? c) A television? d) A non-mobile phone? e) A computer? f) A refrigerator? g) A table? h) A chair? i) A bed with cotton/sponge/spring mattress? j) An electric mitad? k) A kerosene lamp/pressure lamp? | Yes No   a) Electricity…….1 0 b) Radio………….1 0 c) Television………1 0 d) Non-mobile phone.1 0 e) Computer…………1 0 f) Refrigerator………1 0 g) Table………………1 0 h) Chair………………1 0 i) Bed with cotton/sponge/spring mattress………….1 0 j) Electric mitad…….1 0 k) Kerosene lamp/pressure lamp……………….1 0 |  |  |
| 225 | Does any member of this household own:  a) A watch?  b) A mobile phone?  c) A bicycle?  d) A motocycles/scooter?  e) An animal drawn cart?  f) A car/truck?  g) A boat with motor?  h) A bajaja? | Yes No a) Watch . . . . . . . . . . 1 0  b) Mobile phone . . .. 1 0  c) Bicycle. . . . . . . . .... 1 0  d) Motocycles/scooter... 1 0  e) Animal drawn cart . . .1 0  f) Car/truck . . . . . . . . .. .1 0  g) Boat with motor . . .. 1 0  h) Bajaja . . . . . .. ………1 0 |  |  |
| 226 | Does any member of this household have a bank account? | 1. Yes 0. No |  |  |
| 227 | Does any member of this household have a microfinance account? | 1. Yes 0. No |  |  |

Part III: Reproductive history of study participants

| S.no | Questions | Response categories | Skip to | Remark |
| --- | --- | --- | --- | --- |
| 301 | What was your age when you were first married? | 998. I don’t know |  |  |
| 302 | What was your age at your first pregnancy? | 998. I don’t know |  |  |
| 303 | Have you ever become a pregnant? | 0. No 1. Yes | 3 |  |
| 304 | If yes for question 303, how many times? | --------------------------- |  |  |
| 305 | Have you ever faced abortion? | 0. No 1. Yes | 307 |  |
| 306 | If yes for question 305, how many times? | ---------------------------- |  |  |
| 307 | Have you ever delivered a child? | 0. No 1. Yes | 311 |  |
| 308 | If yes for question 306, how many times? | ----------------------------- |  |  |
| 309 | How many of them were live births? | ------------------------------ |  |  |
| 310 | How many of them were delivered at home? | --------------------------- |  |  |
| 311 | Infection during the current pregnancy (e.g., UTI, periodontal disease) | 0. No 1. Yes |  |  |
| 312 | Have you ever experienced death of foetus or stillbirth (fatal death at or after 28 weeks of pregnancy)? | 0. Yes  1. No  998. I don’t remember |  |  |
| 313 | Have you ever experienced death of neonate (age less than seven days)? | 0. Yes  1. No  998. I don’t remember | end |  |
| 314 | If yes to Q 313, how many times? | _____________ |  |  |
| 315 | If yes to Q 313, where was the neonate born? | 1. Home  2. Health institution  3. Other (specify) _________ |  |  |

**Part IV:** Food groups used to calculate dietary diversity score

| Questions | Food group | Food example | Answer |
| --- | --- | --- | --- |
| 1 | Cereals | Corn/maize, rice, wheat, sorghum, millet or any other grains or foods made from these (e.g. Bread, noodles, porridge or other grain products) + insert local foods e.g. Ugali, nshima, porridge or paste | Yes = 1  No = 0 |
| 2 | White roots and tubers | White potatoes, white yam, white cassava, or other foods made from roots |  |
| 3 | Vitamin A rich vegetables and tubers | Pumpkin, carrot, squash, or sweet potato that are orange inside + other locally available vitamin A rich vegetables (e.g. Red sweet pepper) |  |
| 4 | Dark green leafy vegetables | Dark green leafy vegetables, including wild forms + locally available vitamin A rich leaves such as amaranth, cassava leaves, kale, spinach |  |
| 5 | Other vegetables | Other vegetables (e.g. Tomato, onion, eggplant) + other locally available vegetables |  |
| 6 | Vitamin A rich fruits | Ripe mango, cantaloupe, apricot (fresh or dried), ripe papaya, dried peach, and 100% fruit juice made from these + other locally available vitamin A rich fruits |  |
| 7 | Other fruits | Other fruits, including wild fruits and 100% fruit juice made from these |  |
| 8 | Organ meat | Liver, kidney, heart or other organ meats or blood-based foods |  |
| 9 | Flesh meats | Beef, pork, lamb, goat, rabbit, game, chicken, duck, other birds, insects |  |
| 10 | Eggs | Eggs from chicken, duck, guinea fowl or any other egg |  |
| 11 | Fish and seafood | Fresh or dried fish or shellfish |  |
| 12 | Legumes, nuts and seeds | Dried beans, dried peas, lentils, nuts, seeds or foods made from these (eg. Hummus, peanut butter) |  |
| 13 | Milk and milk products | Milk, cheese, yogurt or other milk products |  |
| 14 | Oils and fats | Oil, fats or butter added to food or used for cooking |  |
| 15 | Sweets | Sugar, honey, sweetened soda or sweetened juice drinks, sugary foods such as chocolates, candies, cookies and cakes |  |
| 16 | Spices, condiments, beverages | Spices (black pepper, salt), condiments (soy sauce, hot sauce), coffee, tea, alcoholic beverages |  |

**Part V**: Household food insecurity access scale questions

| 1 | In the past four weeks, did you worry that your household would not have enough food? | 0= No……………………...  1=Yes | Q2 |
| --- | --- | --- | --- |
| 1.a. | How often did this happen? | 1=Rarely (once or twice in the past four weeks)  2= Sometimes (three to ten times in the past four weeks)  3= Often (more than ten times in the past four weeks |  |
| 2 | In past four weeks, were you or any HH member not able to eat the kinds of foods you preferred B/c of a lack of resources? | 0=No…………………….  1=Yes | Q3 |
| 2.a. | How often did this happen? | 1=Rarely  2= Sometimes  3= Often |  |
| 3 | In past four weeks, did you or any HH member must eat a limited variety of foods you B/c of a lack of resources? | 0=No……………………….  1=Yes | Q4 |
| 3.a. | How often did this happen? | 1=Rarely  2= Sometimes  3= Often |  |
| 4 | In past four weeks, did you or any HH member must eat some food that really did not want to eat B/c of a lack of resources to obtain other types of food? | 0= No…………………….  1=Yes | Q5 |
| 4.a. | How often did this happen? | 1=Rarely  2= Sometimes  3= Often |  |
| 5 | In past four weeks, did you or any HH member must eat a smaller meal than you felt you needed B/c there was not enough food? | 0=No………………………  1=Yes | Q6 |
| 5.a. | How often did this happen? | 1=Rarely  2= Sometimes  3= Often |  |
| 6 | In past four weeks, did you or any HH member must eat a fewer meals in a day’s B/c there was not enough food? | 0=No………………………  1=Yes | Q7 |
| 6.a. | How often did this happen? | 1=Rarely  2= Sometimes  3= Often |  |
| 7 | In past four weeks, was there ever no food to eat of any kind in your HH B/c of lack of resource to get food? | 0=No……………………….  1=Yes | Q8 |
| 7.a | How often did this happen? | 1=Rarely  2= Sometimes  3= Often |  |
| 8 | In past four weeks, did you or any HH member go to sleep at night hungry B/c there was not enough food? | 0=No……………………….  1=Yes | Q9 |
| 8.a. | How often did this happen? | 1=Rarely  2= Sometimes  3= Often |  |
| 9 | In past four weeks, did you or any HH member go to a whole day and night without eating anything B/c there was not enough food? | 0 = No……………………….  1=Yes | End |
| 9.a. | How often did this happen? | 1=Rarely  2= Sometimes  3= Often |  |

Part VI: Knowledge on nutrition

| 601 | What is the quantity of food taken during pregnancy? | 0. Less than or the same as before 1. More than before |
| --- | --- | --- |
| 602 | How should a pregnant woman eat in comparison with a non-pregnant woman to provide good nutrition to her baby and help him grow? | 0. Eat less food or energy 1. Eat more food (more energy) |
| 603 | Do you think that weight gain during pregnancy is normal process? | 1. Yes  0. No |
| 604 | Do you think that under-nutrition during pregnancy is genetic origin? | 1. Yes  0. No |
| 605 | Have you heard about under-nutrition during pregnancy? | 1. Yes  0. No |
| 606 | Have you heard about iron-deficiency anaemia? | 1. Yes  0. No |
| 607 | Have you heard about vitamin A deficiency during pregnancy? | 1. Yes  0. No |
| 608 | Have you heard about vitamin B12 deficiency during pregnancy? | 1. Yes  0. No |
| 609 | Do you think inadequate dietary intake and infection cause under-nutrition during pregnancy? | 0. No 1. Yes |
| 610 | Do you think under-nutrition during pregnancy increase risk of complications? | 0. No 1. Yes |
| 611 | Do you think under-nutrition during pregnancy increase risk of bad pregnancy outcomes? | 0. No 1. Yes |
| 612 | Do you think under-nutrition during pregnancy increase risk of death of mother and new-born? | 0. No 1. Yes |
| 613 | Do you think under-nutrition during pregnancy is communicable? | 0. No 1. Yes |
| 614 | When a pregnant woman is undernourished, she is at risk of having a low-birth-weight baby, meaning that the baby is small or has a low birth weight. | 0. No 1. Yes |
| 615 | Most women would benefit from major types of supplements, or tablets, during pregnancy. | 0. No 1. Yes |
| 616 | Do you think under-nutrition during pregnancy can be prevented? | 0. No 1. Yes |
|  | **Part VII: Questions related to attitude on nutrition during pregnancy** |  |
| 701 | How good do you believe it is to eat more food during pregnancy? | 1. Not good at all 2. Not good 3. You’re not sure 4. Good   5. Very good |
| 702 | How difficult do you feel about eating at least one additional meal during pregnancy? | 1. Very difficult  2. Difficult  3. Neutral  4. Easy  5. Very easy |
| 703 | How likely do you feel you are to be iron-deficient/anaemic? | 1.Not likely at all   2. Not likely   3. You’re not sure  4. Likely  5. Very likely |
| 704 | How serious do you feel iron-deficiency/anaemia is? | 2. Not likely at all 3. Not likely   3. You’re not sure  4. Likely  5. Very likely |
| 705 | How good do you believe it is to prepare meals with iron-rich foods such as beef, chicken or liver? | 1. Not good at all 2. Not good 3. You’re not sure 4. Good 5. Very good |
| 706 | How difficult do you feel is it to prepare meals with iron-rich foods? | 1. Very difficult 2. Difficult   3. Neutral  4. Easy  5. Very easy |
| 707 | How confident do you feel in preparing meals with iron-rich foods? | 1. Not confident at all  2. Not confident  3. Medium  4. Confident  5. Very confident |
| 708 | How likely do you feel to have lack of vitamin A in your body? | 3. Not likely at all 4. Not likely   3. You’re not sure  4. Likely  5. Very likely |
| 709 | How serious do you think a lack of vitamin A is? | 1. Not very serious  2. Not serious  3. Neutral  4. Serious  5. Very serious |
| 710 | How much do you like the taste of the following foods? | **Liver** 1. Very dislike 2. Dislike 3. Neutral 4. Like 5. Very like  **Kidney** 1. Very dislike 2. Dislike 3. Neutral 4. Like 5. Very like  **Heart** 1. Very dislike 2. Dislike 3. Neutral 4. Like 5. Very like  **Egg** 1. Very dislike 2. Dislike 3. Neutral 4. Like 5. Very like  **Milk** 1. Very dislike 2. Dislike 3. Neutral 4. Like 5. Very like  **Cheese** 1. Very dislike 2. Dislike 3. Neutral 4. Like 5. Very like  **Yogurt** 1. Very dislike 2. Dislike 3. Neutral 4. Like 5. Very like  **Orange colored vegetables**   4. Very dislike 2. Dislike 3. Neutral 4. Like 5. Very like   **Orange sweet potato**   5. Very dislike 2. Dislike 3. Neutral 4. Like 5. Very like   **Carrot** 1. Very dislike 2. Dislike 3. Neutral 4. Like 5. Very like  **Pumpkin** 1. Very dislike 2. Dislike 3. Neutral 4. Like 5. Very like  **Green vegetables**  1. Very dislike 2. Dislike 3. Neutral 4. Like 5. Very like |
| 711 | How serious do you feel a lack of iodine in the body is? | 1. Not very serious  2. Not serious  3. Neutral  4. Serious  5. Very serious |
| 712 | How good do you feel it is to prepare meals with iodized salt? | 1. Not good at all 2. Not good 3. You’re not sure 4. Good 5. Very good |
| 713 | How difficult is it for you to buy and use iodized salt? | 6. Very difficult 7. Difficult   3. Neutral  4. Easy  5. Very easy |
| 714 | How likely do you feel you are to be under-nutrition during pregnancy? | 1. Not likely at all 2. Not likely 3. You’re not sure 4. Likely   5. Very likely |
| 715 | How serious do you feel under-nutrition during pregnancy is? | 1. Not likely at all 2. Not likely   3. You’re not sure  4. Likely  5. Very likely |
