# Supplementary material for: Effect of amaranth flatbread on maternal anthropometry during pregnancy: Evidence from a cluster-randomized controlled trial in Southern Ethiopia
Source: PLoS One. 2026 Oct 8;21(10):e0360480. doi: 10.1371/journal.pone.0360480 (PMC13648999; doi:10.1371/journal.pone.0360480)
Supplement: Supplementary file 8 [file pone.0360480.s009.doc]

**Project title**(maximum 100 characters)

**Maternal under-nutrition and effect of amaranth grain flat-bread on anemia in northern zone of Sidama region, Ethiopia.**

**Project summary** (maximum 300 words)

**Introduction:** In Ethiopia, maternal malnutrition is a major public health concern. Underweight and anaemia are the two most common nutritional disorders in pregnant women, and they frequently co-exist. A prevalent kind of nutritional anaemia is iron-deficiency anaemia (IDA). These issues are primarily caused by a lack of important micro and macronutrients in the diet, as pregnant women eat primarily cereal-based foods. As a result, the burden of under-nutrition in Ethiopia necessitates a more in-depth investigation of underused crops that are high in nutrients and have the potential to minimize food and nutrition insecurity. Although the amaranth plant grows widely in Ethiopia and can boost food intake, its nutritional potential has yet to be acknowledged by community consumers and health professionals.

**Methods:** A community-based cross-sectional study will be conducted on a sample of 528 pregnant women in their first trimester to examine the prevalence of anemia and identify its risk factors and determine the prevalence of co-existing anemia and under-nutrition among pregnant women. From pregnant women who will be diagnosed for anemia, C-reactive protein (CRP) and serum ferritin will be analyzed to determine IDA. Following this, a parallel-group community-based randomized controlled trial will be conducted for six months among 306 anemic women to evaluate the effect of locally prepared amaranth grain flat-bread on hemoglobin concentration and anemia prevalence compared to the frequently consumed maize bread among pregnant women. A multi-stage sampling method will be utilized to select eligible pregnant women. The households with pregnant women will be identified by conducting the house-to-house census.For categorical variables, summary measures will be presented as absolute frequencies and percentages, but for numerical variables, the mean with standard deviation (SD) will be utilized as a descriptive measure after the distribution is checked for normality. A multi-level mixed-effect logistic/linear regression model will be used to account for between and with cluster effects. We will be used intention-to-treat analyses approach.

**Part 2: Project Description**

**2.1. Personnel & institutional development**

Amanuel Yoseph is a staff member of the Hawassa University College of Medicine and Health Sciences School of Public Health in Ethiopia. I have experience with undernutrition problems among women of reproductive age and underfive children since being the head of the Boricha Health Center and District Health Office in Sidama region, which is an undernutrition hotspot district. I have conducted my masters degree research on the effect of intestinal parasite infection on undernutrition among 6-59-month-old children in Boricha district and published it in the BMC Public Health Journal. Also, I have investigated the determinants of stunting in collaboration with staff since I arrived at Hawassa University using a case-control design. The research outlined in this project would serve as the foundation for much of his academic career development and pave the way for his community service provision in order to achieve his mission of community service. Hawassa University is establishing an excellent nutrition research structure and has been approved as a nutrition research excellence center. They have been conducting National Institutes of Health-funded research for over twenty years, and staff and faculty are proficient in safe practices for working with human beings. However, the chance to address the issue of maternal malnutrition, which is a significant national concern for Ethiopia, would help to establish Hawassa University as a key source of research on this macro- and micronutrient deficiency as well as the significance of nutrition in addressing the issues Ethiopian women face throughout the continuum of care (the first 1000 days). The Hawassa University personnel (Mehretu Belayneh) have all collaborated in Ethiopia and have worked on field research on product development and malnutrition in mothers and children under five. Dr. Lakew Mussie is a well-experienced medical doctor (clinician) in treating and managing severe and moderate acute malnutrition and diseases at Adare General Hospital.Moreover, he is responsible to oversee all laboratory procedure in field settings.

2.2 **Rationale of the project**

Anemia is a global public health concern that affects 36.5% of pregnant women worldwide. This is especially common in sub-Saharan Africa and Southeast Asia . Similarly, 41% of pregnant women are suffering from anemia in Ethiopia, which ranges from 9% in Addis Ababa to 65.9% in the Somali region . Anemia during pregnancy is linked to negative health and socioeconomic outcomes. Pregnant women who are anemic frequently experience decreased physical strength and a higher risk of maternal morbidity and mortality. For instance, severe anemia increases the risk of death by 20% . Anemic pregnant mothers have a higher risk of preterm birth, fetal anemia, low birth weight (LBW), intrauterine fetal growth restriction, decreased gestational weight gain, and perinatal death . Furthermore, anemia can result in a vicious cycle where a person's ability to work is diminished, which can have an adverse economic effect on both the individual and society . Maternal anemia also has an impact on the fetus and baby, such as fetal

The World Health Organization (WHO) designed two broad strategies to address the issues of anemia: nutrition-sensitive and nutrition-specific. Nutrition-specific strategies address the proximal causes of anemia, primarily inadequate dietary intake of hematopoietic nutrients such as iron or vitamin A, supplementation, and access to fortified foods. Dietary diversification is an approach designed to increase the availability, accessibility, and use of locally accessible and acceptable foods that have high micronutrient content and bioavailability across the year . The Ethiopian government has implemented different strategies to prevent anemia among pregnant women, such as promotion of dietary diversification and iron and folic acid supplementation, malaria prevention and treatment, utilization of bed nets, and deworming pills . Also, the government has exerted considerable efforts to decrease maternal anemia by adopting and developing a nutrition and food strategy, program, and policy. Furthermore, the government developed the “Seqota Declaration” to guarantee year-round 100% access to sufficient food in the country by 2030 . Moreover, health extension programs, specifically focusing on community-wide nutrition education, are considered the most significant measures that can help promote adequate nutrition in the first 1000 days .

Despite all national commitments, measures, and efforts, anemia prevalence among pregnant women was high in the country as a whole, particularly in rural areas . Also, there were considerable regional and urban/rural disparities in the prevalence of anemia at the national level , implying the need for further research into the landscape of anemia prevalence patterns in local settings. However, previous studies on the prevalence of anemia among pregnant women in Ethiopia focused primarily on individual-level factors, with little consideration given to community-level and contextual factors. Furthermore, the factors contributing to anemia in pregnant women vary by region in Ethiopia, the prevalence of anemia is highly variable, and existing evidence is insufficient to develop efficient and effective prevention strategies.

Under-nutrition and anemia are two crucial indicators of maternal under-nutrition that can impact the health of both the mother and the fetus. Maternal anemia is linked to an increased risk of preterm birth, stillbirth, LBW, and neonatal deaths. If anemia and underweight co-exist at the same time, the risks increase . However, there is limited evidence on the coexistence of under-nutrition and anemia among pregnant women in developing countries, including Ethiopia, the effect of co-existence on pregnancy outcomes, and whether certain interventions should be targeted toward women who share this dual burden.

Dietary diversity is low in the majority of Ethiopian households. Women's diets are largely comprised of starchy cereals that are poor in micronutrients. Animal-source foods are high in ferrous and other minerals , yet their consumption in the study area has been decreasing due to rapid population growth and unplanned urbanization.

Therefore, it is important to investigate cost-effective approaches to improve the declining nutritional status of Ethiopian women . If amaranth grain were to become widely consumed, it might improve local diets and boost nutrient intake. Amaranth grain is not widely consumed in Ethiopia, where it is even regarded as a wild plant in the southern parts.

The current study is designed to improve nutritional status and households' food security using cost-effective amaranth grains, which are poorly utilized by the communities despite being commonly grown in the study setting. These tactics will benefit governmental organizations, other development allies, and non-governmental organizations to relieve the severe scarcity of foods in developing countries, including Ethiopia. The results from this study will be helpful to inform program managers, policymakers, and implementers in designing effective promotion strategies to improve maternal nutrition status and achieve the sustainable development goals (SDGs). Furthermore, this study will generate results that can inform maternal health champions by providing associated risk factors for anemia and under-nutrition in the context of the Sidama region. Therefore, we aimed to evaluate the effect of amaranth grain flat-bread on the anemia, IDA, and hemoglobin levels of pregnant women and assess the prevalence and associated factors of anemia and co-existing under-nutrition and anemia in the study setting

**Overview of the historical origin, production, and utilization of amaranth**

Amaranth has been grown for over 7, 000 to 8,000 years and first originated in Europe and America, with some nations using it as a staple food. It is a yearly plant with erect growth habits that is grown for vegetables as well as for its seeds, which are utilized as grain .

So far, only South Africa has begun to invest more in extensive studies and ambitious projects, and amaranth is likewise scarce throughout the African continent. However, amaranth's nutritional value and practical qualities have led to a recent surge in its production and use throughout Africa, including Ethiopia. The Kenya Ministry of Agriculture formally registered amaranth as a food crop in Kenya in 1991. To combat poverty, the Kenyan Commission on Poverty Eradication (KCPE) is pushing amaranth as a cash crop . Amaranth production has recently been encouraged in Uganda due to its high productivity and potential for income generation .

In Ethiopia, amaranth is grown as a cash crop in the Benchi Maji region in the country's west. Ethiopia is home to three distinct color-differentiated amaranth varieties: black, red, and white. It is grown either alone or in combination with sorghum or maize three times a year . Though its production is still in its early stages, amaranth is a crop that is largely ignored, particularly in Ethiopia. For instance, the yield of amaranth is 2.25 tons/ha in Ethiopia, which is 17 times less than the global average of 35–40 tons/ha . Amaranths are primarily produced and consumed in the humid regions of Gambella, South Ethiopia, Oromia, and Benashangul Gumuz . However, the Ethiopian government committed to promoting the production and utilization of amaranth and considered it a possible solution to Ethiopia’s food security and nutritional crisis .

In the study area, amaranth grows like a wild plant and is consumed for its grains and leaves during drought seasons. In addition, the grain portion is used in the research region and other regions of the nation for the production of alcoholic beverages as well as for medicinal purposes. However, some volunteer farmers have been producing amaranth, which has been encouraged and started by non-governmental organizations (NGOs).

**Agriculture of amaranth**

Amaranth's exceptional agronomic performance can be attributed to its resistance to pests, heat, and drought. It can grow in any kind of weather, particularly in areas where maize can be grown . Whereas grains can be collected 60–90 days after planting, leaf gathering typically begins approximately four weeks after planting. It can withstand dry conditions because of its rapid growth, which is enhanced by hot temperatures, intense sunlight, and dry soil. Compared to traditional cereals (such as corn, wheat, and rice), grain amaranth requires less fertilizer, water, and energy and can produce greater quantities of product than maize . When properly dried, the grain amaranth has an extended storage shelf life and resists pest infestation .

**The nutritional quality of amaranth grain**

Amaranth is valued for its high levels of protein, minerals, and other nutritional qualities. Research shows that, compared to other cereals, amaranth has remarkably higher levels of protein, iron, zinc, calcium, and soluble and insoluble fiber, though the exact nutritional value will change based on the variety of amaranth and the preparation method . In 100 g of edible amaranth, the seeds contain the following nutrients: lipids 1.56 g (7.5%), 102 calories, carbohydrates 23 g (66.0%), fiber 2.1 g (6.9%), starch 20 g, and protein in its crude form, 11 g to 19 g, comprising the two vital amino acids like methionine and lysine that are not commonly found in other grains . It also compresses 47 mg calcium, 65 mg magnesium, zinc 3.2–2.7 mg/100 g, iron 7.6–18.0 mg/100 g, 6 mg sodium, vitamin C 4.2–4.5 mg/100 g, vitamin B6 0.1 mg, and folate 27.1 mcg . Saturated fatty acids range from 22.4% to 22.8%, while unsaturated fatty acids vary from 76.2% to 77.6% .The health benefits of amaranth grain include helping build and maintain muscle mass, lowering blood cholesterol levels, promoting better heart health, reducing the risk of cardiovascular disease, improving weight and blood sugar control, preventing anemia, increasing antioxidant content, and providing antimicrobial benefits .

### The present value chain of amaranth in Ethiopia and Sidama region

Amaranth has high nutritional and phytochemical profiles that support a host of health benefits and provide defense against a range of chronic illnesses like cancer, diabetes, hypertension, and cardiovascular disorders. It is categorized as pseudo-cereals and is rich in proteins, fats, fiber, vitamins, and minerals, making it highly nutritious. It also has a remarkable ratio of important amino acids . It's also a high-yielding, drought-tolerant, and high-nutrient crop that can be used as ornamentation, animal feed, and human food. This grain is neglected in Ethiopia because of its coarse texture, even though it has many health benefits . Despite its production and consumption in Ethiopia and particularly in the study area, the challenges in production and marketing, as well as the financial advantages to society, have not yet been fully studied, and currently, existing evidence is limited. However, encouraging amaranth value chains is a challenging endeavor, because amaranth is currently a relatively new commodity in Ethiopia with few market outlets. Besides, to assess the effects of producer adoption of enhanced production technologies (better seed varieties) and shifts in amaranth product demand on producer profits and planting behavior, an integrated model of amaranth production and market is needed. Further, improved supply- and demand-side situations necessitate comprehensive, chain-level interventions for the upgrading and commercialization of amaranth value chains. Moreover, production of amaranth is increased by supply-side interventions, but producers receive little financial benefit. In spite of the fact that scientists have long studied and supported underutilized crops, a concentrated and well-coordinated effort is urgently needed to improve the amaranth value chain in Ethiopia. In this context, this study highlights the effect of locally prepared amaranth grain bread on hemoglobin concentration and anemia among pregnant women. Planning innovative research for the effective use of this underutilized grain and ongoing upgrading of the value chain for this crop will greatly benefit from this information.

2.3 **Detailed research plan**
    2.3.1 **Overall aims and objectives of the project**

**Overall aims:** The general objective of this project is to evaluate the effect of amaranth grain flat-bread on anemia, IDA, and hemoglobin levels among pregnant women in the Hawela Lida district of Sidama Region, Ethiopia.

**objectives of the project**

- To assess the prevalence of anemia and identify its associated risk factors among pregnant women.
- To examine the prevalence of co-existing anemia and under-nutrition and associated factors among pregnant women.
- To evaluate the effect of locally prepared amaranth grain flat-bread on hemoglobin concentration, anemia prevalence and maternal anthropometry compared to frequently consumed maize among pregnant women.
  2.3.2 **Hypotheses to be tested**

**H_o_:** The locally prepared amaranth grain flat-bread has no significant effect on the hemoglobin concentration and anemia prevalence compared to the frequently consumed maize bread among pregnant women.

**H_A_:** The locally prepared amaranth grain flat-bread has a significant effect on hemoglobin concentration and anemia prevalence compared to the frequently consumed maize bread among pregnant women.

2.3.3 **Experimental design and methodology**

Study subjects for our study will be chosen from rural districts in the Sidama region of Ethiopia. Subsistence farming is the primary source of income for the study population. Enset (false banana), is their main staple food, followed by maize. Despite the fact that foods in the area may be low in micronutrients and protein, maternal under-nutrition and anemia is a major prevalent problems in the study area . A national survey reported that maternal under-nutrion and anemia are higher in southern Ethiopia, the region in which our study area is located . This population was chosen after careful consideration of the following factors: First, previous studies have shown that maternal under-nutrition and anemia are serious public health concerns in the Sidama region. Second, the study population has not frequently utilized amaranth grain flat-bread, so the effects of the product will be easily detectable. Third, our presence will be acceptable for the community because Hawassa University's research and community service directorate has conducted extensive studies in the field and provided several community services based on the research findings. Lastly, designing sustainable projects that benefit local communities depends heavily on the results of intervention research.

**Amaranth grain flat-bread**

Amaranth grain, maize, and iodide salt will be purchased from the local market or obtained as aid from the local NGOs in the study area. Selected WDT leaders will be trained to bake or produce amaranth grain flat-bread in the intervention arm. The amaranth grain flat-bread will be distributed for 6 months (intervention duration). In another group (comparator arm), maize bread will be distributed for six months.

**Study area:** This study will be conducted in the Hawela Lida district of the Sidama region of Ethiopia. Hawela district is one of the 30 districts in the Sidama region and is located 289 km away from Addis Ababa. It has 15 rural and 4 kebeles (the lowest administrative unit in Ethiopia). Based on the Sidama Regional State Health Burea 2022 Report, the district has an overall population of 129,949 with an estimated total of 23,928 households. Of these, 24.30% were women of reproductive age (WRA) . Farming, or agriculture, is the major share of income-producing activity in all kebeles. The main crops grown in the district are enset, maize, coffee, khat, barely, haricot beans, corns, sweet potatoes, and local varieties of cabbage. The district administration has a total of 425 health professionals of different disciplines, 20 health posts, and 4 health centers owned by the government; additionally, there are 5 private medium and two NGO clinics, and 6 private drug stores. The health post is managed by health extension workers (HEWs) and provides Maternal Newborn and Child Health (MNCH) services such as health education, nutritional screening, and treatment for children, pregnant and lactating women, family planning, etc. . The potential health service coverage of the district by health facilities was 70% . The district was chosen based on concern for access to transportation and a good geographical location to supervise and increase the chance to handle several potential challenges that arise during the intervention implementation.

**Study design and period:** A community-based cross-sectional survey will be conducted from April to May 2024 for objectives I and II. A parallel-group, two-arm cluster randomized control trial (cRCT) will be implemented for objective III. The hallmark of this design is that each cluster remains in the arm it was randomly assigned to during the course of the whole study period. Thus, all study subjects are randomly assigned into a group, and all the study subjects in the assigned group obtain or do not obtain an intervention. Study time will follow immediately after studies I and II. Thus, this study will be carried out from July to December 2024.

**Study population:** The study population will consist of all randomly selected pregnant women less than 3 months of gestational age and residing in the district for at least 6 months.

**Inclusion and exclusion criteria:** All randomly selected pregnant women less than 3 months of gestational age and residing in the district for at least 6 months will be included in this study. Women who have a severe illness during the data collection period will be excluded from this study due to their inability to communicate. Also, women who have a psychiatric disorder will be excluded because that could affect participation and consent procedures. Besides, pregnant women who have plan to change residence during the implementation of an intervention will be excluded for objective III because this will lead to a loss of follow-up. Pregnant women will be excluded from the study if they have chronic diseases like tuberculosis, HIV/AIDS, cancer, , have had a blood transfusion in the last 6 months, have had malaria at least 3 times in the last 3 months, or are unwilling to participate. Besides, pregnant women with severe anemia (<7 g/dl) detected during the survey will be excluded from the trial and referred to a nearby health facility for medical treatment. All pregnant women in both arms will be provided piperazine citrate syprup 750 mg/30 ml stat to treat soil transmitted helmentiasis regardless of their parasitic infection status

**Sample size calculation:** The sample size is computed using OpenEpi version 3 for objectives I and II. The sample size required to estimate the prevalence of anemia is computed by considering the anticipated prevalence of IDA (19.3%), according to the report of a previous study , a margin of error of 5%, a 95% confidence level, and a design effect of 2. The design effect is calculated using the formula DEFF = 1+ (n-1)*ICC, where 'n' is the average cluster size and ICC is the intraclass correlation coefficient. The minimum required number of clusters is computed by multiplying the effective sample size by the ICC. However, previous studies didn’t report the ICC value. We took the typical value of the ICC to be 0.05 from a range of values (0.01 to 0.05) based on the recommendation of Donner . Accordingly, the minimum needed number of clusters is 240*0.05 = 12. In this study, *kebeles,* which are subsets of districts, were considered clusters.The sample size is adjusted for the non-response rate by adding 10% of the anticipated non-response rate. Based on the above information, the estimated sample size is 528. Hence, the sample size of 528 obtained from the first objective is utilized because it is the maximum sample size determined and would be adequate to address objectives I and II.

The sample size is calculated using OpenEpi version 3.01 and based on the following considerations for objective III: Due to the lack of a previous cRCT on the same topic, assumptions on the proportion of pregnant women with a prevalence of IDA in the control are taken from a mini-2019 Ethiopian Demographic and Health Survey . The national prevalence of IDA is taken as the prevalence of IDA in the control arm, and we assumed the prevalence of IDA among pregnant women would be decrease by -10% after the intervention in the intervention arm. Accordingly, P1 = 41.0% (proportion of women with IDA during pregnancy) in the control group, and P2 = 31.0% (proportion of women with IDA during pregnancy in the intervention arm), 95% CI, and 80% power. According to the above considerations, the estimated effective sample size for individual-based randomization is 102in each arm. The minimum required number of clusters is computed by multiplying both groups' effective sample size and ICC factors . We received the typical value of the ICC factor of 0.05 from the range of values (0.01 to 0.05) based on the recommendations . Consequently, the minimum needed cluster number is 204*0.05 = 10.2 for both groups. Nevertheless, to maintain the cluster's sufficiency and adequate power , 12 clusters will be included in this study. To account for the effect of the cluster, the effective sample size is multiplied by a variance inflation factor (VIF) of 1.5. The VIF is calculated assuming an equal cluster size of 17 study subjects from 12 clusters. We used an ICC value of 0.05 (VIF = 1+ [(n-1) ICC]), where 'n' is the average cluster size . Thus, the final estimated sample size is 306 (153 in the intervention arm and 153 in the control arm).

**Sampling technique:** Initially, the determined sample size will be proportionally allocated to the kebeles in the district based on their population size. A multi-stage sampling method will be utilized to select the representative study participants until the determined sample size is reached. The first stage will be the selection of Hawela Lida district from the Sidama region using a purposive sampling method. In the second stage, representative kebeles will be selected from the district using a simple random sampling method. Based on the section procedure, 12 kebeles, namely Hawela Lida 01, Murancho, Dobe, Nagasha, Chafe 01, Galuko, Hireye, Gara, Galo, Tewerako, Chafe, and Lida, were randomly selected from the district. The third stage will be a selection of households with pregnant women. The households with pregnant women will be identified by conducting a house-to-house census, and a sampling frame will be prepared. Finally, study respondents will be drawn using a simple random sampling technique. If the selected woman is absent from the household for three successive visits during the data collection period, she will be considered a non-respondent. One mother will be included by using a simple random sampling procedure when two or more mothers occur in the selected households.

**Randomization:** The study site consists of 19 kebeles. For this study, these kebeles will be stratified into two strata based on the place of residence, such as rural and urban kebeles. Stratification based on location aims to reduce stratum variation and helps to ensure that the covariates are balanced between two arms . To increase the resemblance between the two arms, a similar number of clusters from each stratum will be allocated to both groups. Hence, four urban kebeles from the district will be allocated to each group by using the lottery method of simple random sampling. Similarly, 15 rural kebeles from the district will be allocated to each arm by using the lottery method. Lastly, 26 study subjects from each cluster will be randomly selected and included in the study.

**Intervention and trial groups:** The pregnant women in the control and experimental groups will be referred to as the "maize arm" and the "amaranth arm," respectively. Pregnant women in the intervention arm (N = 153) will receive 200 g of flat-bread on a daily basis, comprising 30% chickpea and 70% amaranth grain in mass, for six months. Pregnant women in the control arm (N = 126) will receive 200 g of bread on a daily basis, comprising 100% maize, for six months.

**Recipe preparation:** The recipe will be produced according to the recommended dietary allowance (RDA); based on RDA, 200 g of flat-bread (30% chickpea and 70% amaranth grain) comprised 30 mg of iron, which can satisfy 50% of RDA supposing 15-20% iron absorption . a study from the Hawassa University Department of Food Sciences reported that amaranth-grain flat-bread was safe for consumption for 48 hours after preparation and improved the nutritional values . To lower the phytate levels in the amaranth grain, home-level processing will be used. For 24 hours, amaranth grain will be soaked in water with 5 milliliters of lemon juice per 100 milliliters of water, and it will germinate for 72 hours. It will be sun-dried, roasted, and ground using a nearby electrical mill before fermented bread is made . In order to achieve the desired color and structure similarity to amaranth bread, maize will also be roasted and fermented at the same time as the amaranth grain. To support our procedures, studies done in Ethiopia and Kenya showed that homemade processing of amaranth grain, such as soaking and germination, was found to significantly reduce phytate levels and increase iron and other macro- and micronutrient bioavailability . We will send a 200-gram sample of processed amaranth grain to an Ethiopian public health institute laboratory to determine the iron bioavailability of the processed amaranth grain bread. The laboratory technologists will report the amount of iron and phytate that are found in our sample by using milligrams. We will convert the reported amount from milligrams to grams by dividing the reported amount of iron and phytate by 1000. At this stage, we have the iron and phytate contents of processed amaranth grain. We will use a formula to **determine the molar ratio of phytate to iron** (bioavailability). **T**he molecular weight (mole) of phytate and iron will be determined by dividing the weights of phytate and iron by their atomic weights (phytate: 660 g/mol and iron: 56 g/mol). The molar ratio between phytate and iron will be obtained after dividing the mole of phytate with the mole of minerals. The critical molar ratio, above which ion absorption may be impaired, is phytate: iron >14 (32). Depending on the values from the molar ratios, the bioavailability of the iron will be judged.

**Acceptability testing:** An acceptability study will be conducted in Boricha district of Sidama region (the adjust district which have similar context and composition of pregnant women) to determine the acceptability of the prepared amaranth grain and maize bread before the intervention. Acceptability testing will be conducted following a protocol modeled on that of the previous study . The purpose will be to determine the taste, acceptability, and physical tolerance of the newly prepared amaranth grain and maize bread. First, we will test the locally prepared amaranth grain and maize bread at the Hawassa University staff level based on trial proportion. Then, pregnant women will be identified at households in Boricha district and randomly assigned to locally prepare amaranth grain and maize bread, which will be prepared at the laboratory trial. Feeding will be directly observed at the site, and the time it took for the pregnant women to consume the entire serving of bread will be measured, as well as the amount of bread remaining if not completely consumed. Pregnant women will be asked to estimate the locally prepared amaranth grain and maize bread palatability and overall likability on a 5-point hedonic scale that graphically illustrates a series of human faces with varying degrees of smile or discontent. Bread distributers will then be provided the bread to continue daily feeding at home and go home on day 4 to report again on the pregnant women’s tolerance of the bread and any adverse reactions, including diarrhea and allergy.

The locally prepared amaranth grain and maize bread selected from this sensory evaluation will be further subjected to the pregnant women for the first two weeks of study by trained WDA. At the end of the second week, an exit interview will be conducted to assess perceptions, utilization, level of acceptability, and possible intra-household distribution of the product, as well as the ease and difficulty of providing the bread. The acceptability will increase substantially over time, and pregnant women will be happy to feed the food from plant sources during pregnancy in Ethiopia due to cultural taboos of animal origin .

Pregnant women and the research team will be informed of the benefits of the locally prepared amaranth grain and how those could improve the nutritional status of their pregnant women. A demonstration will be given on the preparation of the locally prepared amaranth grain bread for WDT leaders. The main components will be amaranth, maize powder, chickpeas, iodide salt, and cooking oil. Detailed counseling will be given, emphasizing specific messages for increasing the portion size, methods of preparation of the locally prepared amaranth grain bread, storage, hygiene practices, and serving to ensure safe food handling and consumption for WDT leaders. One of the key messages states that locally prepared amaranth-grain bread is a food and medicine for mildly and moderately sick pregnant women and is not to be shared with other family members. Pregnant women will be advised to prepare locally prepared amaranth grain bread from their own resources and feed themselves and their families. After the intervention is completed, the community and pregnant women will be advised to adopt the rehabilitated mild and moderate anemia in their normal family setup.

**Masking and bread distribution:** The trial subjects, bread distributors, and outcome assessors (data collectors) will be all unaware of the study groups assignment and bread content. The bread distributers for the maize and amaranth groups will be distinct, and there will be 12 bread-distributers, one for each *kebele*. Twelve boxes, one for each *kebele,* will be packed and tagged with the name of the pregnant women. Every day, each bread-distributer fed the pregnant women at their home, under their close supervision. Unopened bread will be returned and recorded by the coordinator each day in the event of absences or refusals.

**Monitoring compliance of intervention:** the bread distributors will be assessed the compliance of the pregnant women at their home, under their direct supervision. They will be reported to the coordinator any issues like refusals, non-compliance, and protocol deviation and the coordinator will be recorded in the protocol adherence-registration-books.

**Intervention research team training**

Two WDT leaders will receive training on how to prepare flour at home using home processing methods (germinating, soaking, drying, roasting, and milling), one for maize flour and the other for amaranth flour. They will also receive training in bread packing, cooking times, and mixing ratios. Twelve bread distributors who pick up bread from bread producers will receive training on how to inspect packages. They will also receive training on how to feed and serve the women in their homes on a daily basis, providing them with bread.

**Study variables:** The outcome variables are the prevalence of anemia and under-nutrition. In pregnancy, anemia is defined as Hemoglobin <11 g/dl and is classified into three categories based on WHO criteria: mild (10.0–10.9 g/dl), moderate (7.0–9.9 g/dl), and severe (7.0 g/dl) . Hemoglobin analysis will be performed by laboratory technologists in all health posts. Each pregnant woman's hemoglobin concentration will be determined by taking a finger-prick blood sample with a HemoCue Hb 301 (HemoCue AB, Angelholm, Sweden). The site will be disinfected, and a prick will be conducted on the tip of the middle finger. The device used to measure hemoglobin concentration will eliminate the first drop of blood and collect the second drop to fill the microcuvette. The cuvette holder held the microcuvette. To improve test reliability, the meter's performance will be examined daily using control standards. While the meter will be prepared to utilize capillary blood, the microcuvette owner will be first pulled to the loading position, and then the sample will be filled continuously for the examination. Within 10 minutes of filling, it will be placed in the holder and pushed into its measuring position. Finally, using WHO's field survey recommendation techniques, the result will be recorded and displayed after a duration of 15–60 seconds . Before the data is entered, the hemoglobin level will be corrected for altitude using the formula . Serum ferritin and C-reactive protein will be tested further in all samples with hemoglobin levels less than 11 g/dl. The Cobas 6000 e601 module from Roche (Germany) will be used to analyze serum ferritin, and the Cobas 6000 c501 module will be used to analyze CRP. All laboratory procedures will be carried out in accordance with standard operating procedures (SOPs). In this study, the MUAC measurements of pregnant mothers who fall below a cutoff point of 23 cm will be classified as undernourished, while those who measure 23 cm or more will be classified as normal. A flexible, non-stretchable tape will be used to measure the mothers' MUAC by placing them in the Frankfurt plane and observing them sideways to measure the left side arm to the nearest 0.1 cm. The mothers' arms will be hanging lightly at the side with their palms facing inward.

The independent variables are classified into individual and community-level covariates. The individual-level covariates are socio-economic and demographic covariates like women age, occupation of the women and their husbands, education of the women and their husbands, wealth index, use of mass media and health insurance enrollment status; obstetric characteristics such as women age at first marriage and childbirth, gravidity and parity status, the experience of obstetric danger sign, history of stillbirth, and pregnancy planning status; knowledge and practice of dietary diversity; household food security and water, sanitation and hygiene status. The community-level covariates are place of residence, community-level distance from the nearest HF, community-level literacy of women, community-level social media use and community-level poverty. The intervention or exposure variable is health education. The intervention group will receive locally prepared amaranth grain bread for six months until the date of delivery, while the comparator group will receive the frequently consumed maize bread.

Dietary diversity scores for women will be calculated using food and agriculture organization (FAO) guidelines . After computation, the score will be divided into three tertiles: low, medium, and high. Nine food groups will be created from all of the pregnant women's reported foods and drinks consumed the day before the survey: cereals and starchy staples; oils and fats; dark green leafy vegetables and vitamin A-rich fruits and vegetables; legumes; nuts and seeds; other fruits and vegetables; meat and fish; organ meat; milk and products; and eggs. Pregnant women who have eaten the food in each subgroup (at least once) will receive a score of 1, and otherwise, 0 will be assigned.

The household food security questionnaires are based on food and nutritional technical assistance (FANTA) version 3 and have been modified for the local context; they contain 18 questions . The first nine questions are answered "yes" or "no," and the results are divided into four groups: food secured, mildly food insecure, moderately food insecure, and severely food insecure.

**Data collection tool and procedure:** The study tool is a structured and pretested interviewer-administrated questionnaire developed from similar previous studies . The questionnaire is initially prepared in English. It is translated into the Sidaamu Afoo language (the prime language spoken by almost all of the study respondents) and reconverted back to English to maintain its originality and consistency. The translation will be conducted by a language expert in both English and Sidaamu Afoo. The translated study tool will be reviewed by the principle investigator (PI) and a third person who is also an expert in both languages. Then, any inconsistency or non-accuracy between the two versions will be corrected based on the problems noticed during the review.The data collection procedure will be managed by 12 data collectors and 2 supervisors. Data collection will be conducted, and the PI will monitor and control the overall process of data collection and make appropriate corrections for any issues.

**Blood collection and serum preparation procedures**

Standard procedures will be followed for blood sample collection and laboratory analyses. Phlebotomists will prepare the necessary materials (alcohol swabs, sterile gloves, a tourniquet, a vacutainer needle, a vacutainer tube, and a syringe) and collect blood from each participant. They will clean and dry the collection site, apply a tourniquet, locate the vein, and prepare the needle and tube. Participants will be instructed to make a fist, and the phlebotomist will insert the needle to collect 5 ml of blood. Afterward, the needle will be withdrawn, pressure will be applied to the puncture site, and the needle will be safely disposed of. The phlebotomist will express gratitude to the participants for their cooperation.

After blood collection, the hemoglobin concentration will be measured in the field using a Hemocue photometer (Hb 301 + System) with collected blood drops. Altitude adjustments for hemoglobin will be made based on WHO standards. To prevent hemolysis and contamination, serum extraction will occur within 45 minutes of sample collection at the nearby health institution. The extracted serum samples will then be frozen at -20 °C and transported on dry ice to the Hawassa Referral Comprehensive Specialized Hospital for further analysis.

Serum ferritin levels will be analyzed using the Elecsys 2010 analyzer, an automated clinical analyzer manufactured by Elecsys in Mannheim, Germany. The analysis will utilize the enzyme-linked immunosorbent assay (ELISA) method. Additionally, serum C-reactive protein (CPR) levels, used to assess inflammation, will be measured using the Cobas 6000 system, a clinical chemistry analyzer manufactured by Roche Diagnostic GmbH.

**Data quality assurance:** The training regarding study tools will be provided for the data collectors and supervisors by the PI for 2 days. During the training, attention will be given to the significance of the study, data collection procedure, objectives, methods, and ethical issues. The data collection will be conducted using a properly designed, standardized, pretested, structured, face-to-face interviewer-administered questionnaire. After the pre-test, essential adjustments will be conducted before the actual data collection process. The data collection process will be carefully supervised. A consistent checkup for incompleteness and non-consistency of the data will be made daily. The data will be cleaned, coded, and exported to SPSS version 26 and Stata 17 for analysis. The incomplete, inconsistent, and invalid data will be distinguished correctly to acquire high-quality data before, during, and after data analysis. To minimize the risk of reporting bias, data collectors will be told during the training to properly explain to the study participants the importance of the questions and to elicit a genuine response from the participants. In addition, to minimize the risk of bias, maximum efforts will be taken by careful selection of subjects that represent the source population, maximizing response and follow-up rates, provision of training for the data collectors and supervisors, double masking, and randomization. The randomization will be done by an autonomous, blinded statistician using an SPSS random number generator. The data collectors after the intervention remain blinded to intervention groups. Training will be provided to two medical laboratory technicians on blood collection techniques, hemoglobin analysis techniques, and how to prepare and send blood serum to the regional laboratory center. The serum ferritin and CRP levels will be examined by two laboratory professionals at the national level.

**Auditing:** This study is open for auditing via any legitimate concerned body or organization, such as governmental and non-governmental organizations, as necessary.

**Protocol modification:** Timely communication will be conducted about the study protocol amendment with the concerned bodies, like the Institutional Review Board (IRB) and project sponsors or funders.

**Dissemination of the findings:** The findings of the study will be published in peer-reviewed international journals and discussed at national and international seminars and scientific conferences. Also, the findings will be officially presented to the Hawassa University School of Public Health. The summary of the main findings will be delivered to the district health office and the Sidama region health bureau. The findings will also be distributed to vital stakeholders in maternal health policy, including the WHO.

**Protocol reporting guidelines:** The primary outcomes of this study will be reported based on the CONSORT 2010 statement: extension to cluster randomized trials guidelines. The secondary outcomes will be prepared based on the Strengthening the Reporting of Observational Studies in Epidemiology (STROBE) guideline.

**Trial regresitration:**The trials proposed in this protocol will be registered at ClinicalTrials.gov once ethical approval is obtained.

**Ethical consideration:** The ethical approval of this protocol will be obtained from the IRB at the College of Medicine and Health Sciences of Hawassa University. A support letter will be obtained from Hawassa University School of Public Health, Sidama Region Health Bureau, and kebele administrators. The two levels of consent will be required before conducting the actual study. First, community leaders must approve on behalf of the community before randomization. Second, written consent will be obtained from study participants. The aim of the study and methods of data collection, privacy, voluntary participation, potential benefits, and harms will be described to the study participants before they sign informed written consent. Written informed consent will be received from all study participants who meet inclusion criteria before enrollment. All data collection methods and intervention techniques will be carried out with confidentiality. During the data collection and storage, the confidentiality of the data will be assured. Specific personal identifiers will not be collected, and researchers will not have access to data that could recognize individual respondents during or after data collection. The maximum efforts will be exerted to register the protocol in the international community trials registry.There is no risk or harm in participating in this study except for 1 hour of consumption per month. There may be a slight discomfort when sharing some personal information. However, we do not wish this to happen, and you may refuse to answer any of the questions if you feel uncomfortable.

2.3.4 **Data analysis strategies**

During the analysis, quantitative variables will be handled by recoding, calculations, and categorizations before carrying out the main analysis. Descriptive analyses will be carried out to get descriptive measures for the important variables of interest. The Principal Component Analysis (PCA) will be carried out for the computation of the wealth index .

This analysis will include all women included in the trial at the beginning who utilized at least one amaranth grain bread. An intention-to-treat analysis (ITTA) will be done to compare outcomes between the two groups. A chi-square test will be used to test the intervention effect for unadjusted analysis for categorical outcomes, while an independent t-test will be utilized for numeric outcomes.

Before conducting a multilevel analysis, we will decide whether or not the multilevel model is required by doing a random intercept model of logistic regression. This model offers data regarding ICC . If the ICC value is greater than 5%, the multilevel analysis model is mandatory to consider. Both bi-variable and multivariable analyses using multilevel mixed effects modified Poisson models with random effects will be carried out if the outcome variable prevalence is greater than 20%. If the prevalence is less than 20%, a multilevel mixed effects binary logistic regression model will be utilized to adjust for between- and within-cluster variation for adjusted analysis. Those variables with p-values < 0.25 on the bi-variable analysis and practical significance backed from relevant literature will be included in a multivariable regression model to find out determinants independently associated with anemia and under-nutrition adjusting for other variables in the model . Effect modification will be examined using stratification and entering effect modifiers into the multivariable analysis model one at a time. Multicollinearity among the independent variables will also be evaluated using a multiple linear regression model. The variance inflation factor (VIF) < 5 will be used to declare the absence of multicollinearity .

The presence and strength of a statistically significant association between anemia and the independent variables will be assessed using adjusted odds ratios (AORs) with a 95% CI. A statistically significant association between the variables of interest will be confirmed when the 95% CI of the AORs does not contain 1. All analyses of the study will be carried out using a 95% CI or 5% level of significance.

**2.3.5. Research Institution**

Through research, training, and public services, Hawassa University (HU) aims to contribute to the nation's development by advancing knowledge, enhancing technology creation and transfer, promoting effective entrepreneurship, and instilling a responsible and constitutional attitude.The mission of HU is to become a globally renowned center of excellence in teaching, research, and public service.

**References**
